# Supplementary material for: The VOICE-DEP study protocol: Multimodal analysis of voice and speech during medical interviews to support diagnosis and longitudinal monitoring of major depressive disorder
Source: PLoS One. 2026 Sep 10;21(9):e0353827. doi: 10.1371/journal.pone.0353827 (PMC13561378; doi:10.1371/journal.pone.0353827)
Supplement: Supplementary file 1 [file pone.0353827.s002.docx]

**PROTOCOL**

1. **DESCRIPTIVE TITLE AND PROTOCOL VERSION.**

MULTIMODAL ANALYSIS OF VOICE AND SPEECH DURING THE MEDICAL INTERVIEW AS A TOOL TO SUPPORT DIAGNOSIS AND EVALUATION OF RESPONSE TO TREATMENT IN DEPRESSIVE DISORDER.

**Short title:** VOICE-DEP Study: depression assessment assisted by voice analysis. Version 2, dated 27 April 2026.

2. **STUDY SPONSORS:**

CLINICA UNIVERSIDAD DE NAVARRA

Avenida Pio XII, 36 31008 Pamplona

NNBi 2020 S.L

Pol. Noain Esquiroz - calle H no. 2 office p1 12-13 - 31191 Esquiroz, Navarra

A company specialized in the analysis and interpretation of health data, developing customized artificial intelligence solutions and predictive models (Machine Learning) applied mainly to clinical data.

# 3. RESEARCH TEAM

Principal Investigator: Patricio Molero Santos. Doctor of Medicine. Specialist in Psychiatry.

Clinica Universidad de Navarra.

Azucena Aldaz Pastor. Doctor of Pharmacy. Specialist in Hospital Pharmacy. Clinica Universidad de Navarra.

Maialen Zabalza Zudaire, Graduate in Mathematics and University Master's Degree in Artificial Intelligence. Technical Management and R&D; Specialist. NNBi 2020 S.L.

Onintza Sayar Beristain, Doctor of Pharmacy, promoter and Managing Director of NNBi 2020

S.L.

Felipe Ortuno Sanchez Pedreno. Doctor of Medicine. Specialist in Psychiatry. Clinica Universidad de Navarra.

Paula Fructos Cordovilla. Medical Resident in Psychiatry. Clinica Universidad de Navarra. Franco Fabricio Carpio Chaname. Medical Resident in Psychiatry. Clinica Universidad de Navarra.

Esmeralda Garcia Perez. Medical Resident in Psychiatry. Clinica Universidad de Navarra.

Alvaro Ortiz Agudo. Medical Resident in Psychiatry. Clinica Universidad de Navarra.

Favio Emanuel Nunez Ortiz. Medical Resident in Psychiatry. Clinica Universidad de Navarra.

**4. SUMMARY.**

a) Title: Multimodal analysis of voice and speech during the medical interview as a tool to support diagnosis and evaluation of response to treatment in depressive disorder.

**Short title:** VOICE-DEP Study: depression assessment assisted by voice analysis. PI: Patricio Molero. Clinica Universidad de Navarra

b) Rationale and context.

Major depressive disorder (MDD) is a serious, recurrent and disabling disease with high prevalence in our setting. It is associated with increased morbidity and mortality due to suicide risk and other associated general medical and specifically psychiatric causes, and it is one of the most important causes of disability-adjusted life years lost. Early diagnosis is a key factor for symptom control, functional improvement and prevention of complications. Preliminary evidence suggests that voice analysis may be considered a potential digital biomarker with complementary clinical utility in the diagnosis of depression. The overall objective of this study is to develop and formalize a standardized, reproducible and clinically grounded protocol for the detection and longitudinal monitoring of major depressive disorder using speech-derived digital biomarkers, integrating acoustic, paralinguistic and linguistic features anchored to validated clinical scales, in order to advance research on diagnosis and follow-up of depression assisted by voice analysis.

This is an exploratory pilot study of methodological validity within the context of an industrial doctorate.

c) Research hypothesis and objectives.

Working hypothesis:

Major depressive disorder and its clinical course are associated with changes in certain acoustic, paralinguistic and linguistic characteristics of voice and speech that can be analyzed in a standardized and reproducible way in routine clinical practice.

Primary objective:

To establish a reproducible clinical framework for extracting and analyzing acoustic, paralinguistic and linguistic features from semi-structured clinical interviews, enabling discrimination between patients with a medical diagnosis of major depressive disorder and healthy controls, using voice-based models anchored to validated clinical reference standards (validated symptom-intensity scores objectified by the physician).

Secondary objectives:

1. To build a longitudinal Spanish-language clinical speech corpus comprising repeated voicerecordings from patients with major depressive disorder and single-session recordings from healthy controls.
2. To characterize the relationship between speech-derived digital biomarkers andphysician-rated measures of depression severity, including their evolution over time.
3. To assess the added value of multimodal approaches combining acoustic, paralinguistic andlinguistic features compared with unimodal models.
4. To evaluate intra-individual vocal changes across multiple assessment time points and theirassociation with changes in clinical severity.
5. To provide a transparent methodological framework that may facilitate future replication, cross-comparison and external validation in other clinical presentations and other diagnoses, languages and sociocultural settings.
6. To explore whether any treatment modality is more strongly associated with voice changesand clinical evolution.

d) Study design.

Observational, prospective longitudinal study with a control group.

e) Population.

Patients with MDD followed in the Department of Psychiatry of Clinica Universidad de

Navarra, both in outpatient consultation and inpatient care, regardless of their treatment (psychotherapeutic, pharmacological or neurostimulation, or even patients who decide not to receive treatment but remain under watchful follow-up), with the following inclusion criteria and no exclusion criteria:

# Inclusion/exclusion criteria for patients Inclusion

1. Patients aged 18 years or older,
2. Spanish-speaking (native speakers or with sufficient fluency to conduct a clinical interview)
3. Able to understand the study procedures and provide written informed consent, in the investigator's judgment.
4. With a medical diagnosis of moderate or severe unipolar depression (with or without psychotic symptoms) according to ICD-10 diagnostic categories (Moderate depressive episode (F32.1) or severe depressive episode (F32.2 and F32.3), Recurrent depressive disorder, current episode moderate (F33.1) or severe (F33.2 and F33.3)), regardless of their treatment (psychotherapeutic, pharmacological or neurostimulation, or decision not to receive treatment).

# Exclusion

1. Severe neurological disorders affecting speech production or cognitive function (such assevere dementia). Non-severe neurological disorders or non-severe dementias are allowed.
2. Severe voice or speech disorders unrelated to depression that prevent the clinical interviewfrom being conducted through verbal language.
3. State of intoxication due to substance use at the time of assessment.
4. Other serious medical conditions that interfere with participation in this study or withspeech production.

# Inclusion/exclusion criteria for controls

# Inclusion

1. Age 18 years or older,

Exclusion

1. Personal history of depression or any other psychiatric disorder.
2. Severe neurological disorders affecting speech production or cognitive function (such assevere dementia). Non-severe neurological disorders or non-severe dementias are allowed.
3. Severe voice or speech disorders unrelated to depression that prevent the clinical interviewfrom being conducted through verbal language.
4. State of intoxication due to substance use at the time of assessment.
5. Other serious medical conditions that interfere with participation in this study or withspeech production.

f) Variables.

- Medical diagnosis of moderate or severe unipolar depression (with or without psychoticsymptoms) according to ICD-10 diagnostic categories (Moderate depressive episode (F32.1) or severe depressive episode (F32.2 and F32.3), Recurrent depressive disorder, current episode moderate (F33.1) or severe (F33.2 and F33.3)), regardless of treatment (psychotherapeutic, pharmacological or neurostimulation, or decision not to receive treatment).
- Variables from the multimodal analysis of voice and speech (acoustic, paralinguistic andlinguistic, specified in the variables section).
- Intensity of depressive symptoms objectified by the physician using the Montgomery-AsbergDepression Rating Scale (MADRS) (Montgomery and Asberg 1979; Lobo et al. 2002) (clinical scale completed by the physician), taken from the Basic Instruments Bank for Clinical Psychiatry Practice (Garcia-Portilla et al. 2022).
- Global clinical severity objectified by the physician using the Clinical Global Impression scale(CGI) (Guy 1976) (clinical scale completed by the physician), taken from the Basic Instruments Bank for Clinical Psychiatry Practice (Garcia-Portilla et al. 2022).
- Severity of depressive symptoms reported by the patient using the Patient Health Questionnaire - 9 (PHQ-9) (self-administered questionnaire) taken from the Basic Instruments Bank for Clinical Psychiatry Practice (Garcia-Portilla et al. 2022).
- General medical and psychiatric comorbidities (specified in the variables section).
- Anthropometric, demographic and lifestyle variables (specified in the variables section).
- Pharmacological treatment variables (specified in the variables section).
- Non-pharmacological treatment variables (specified in the variables section).
- Other variables (specified in the variables section).

g) Data sources.

Information on the variables for each participant will be collected at each visit in a pseudonymized case report form.

h) Study sample size.

The planned sample comprises a total of 175 voice recordings. Twenty-five patients with a clinical diagnosis of major depressive disorder will be recorded at 5 longitudinal time points (Visit 0 and 4 monthly visits), for a total of 125 longitudinal records. In addition, 50 healthy control participants will be recorded with a single baseline voice record (Visit 0). Justification is provided in the sample size section.

i) Data analysis.

Statistical analyses and modeling strategy are specified in the Data Analysis section.

j) Stages and schedule.

| **Activity** | **Start date** | **End date** |
| --- | --- | --- |
| Data collection | May 2026 | December 2027 |
| Analysis and interpretation of the data obtained | January 2028 | February 2028 |
| Writing | March 2028 | June 2028 |
| Final results report | September 2028 |  |

# 5. MODIFICATIONS AND UPDATES

Any substantial amendment to the study protocol after the start of data collection, with its justification, date and indication of the protocol section affected.

# 6. STAGES

Table with the planned schedule for at least the following stages:

a) Start of data collection.

b) End of data collection.
c) Study status reports, if applicable. (Not applicable).
d) Interim reports of study results, if applicable. (Not applicable).
e) Final report of study results.

| **Activity** | **Start date** | **End date** |
| --- | --- | --- |
| Data collection | May 2026 | December 2027 |
| Analysis and interpretation of the data obtained | January 2028 | February 2028 |
| Writing | March 2028 | June 2028 |
| Final results report | September 2028 |  |

**7. RATIONALE AND CONTEXT.**

Depression (considered in this project according to the diagnostic criteria of the categories of moderate or severe depressive episodes under ICD-10 (World Health Organization 2016) and major depressive disorder (MDD) under DSM-5 (American Psychiatric Association 2013)) is a serious, recurrent and disabling disease, with high prevalence in our setting (with estimated average lifetime prevalence of 14.6% (Merikangas and Rihmer 2017), and overall prevalence in Europe of around 6-7% (Arias-de la Torre et al. 2023)), causing impairment of general medical health, socio-family functioning, work performance and quality of life. It is associated with increased morbidity and mortality due to suicide risk and other associated general medical and specific psychiatric causes (Akiskal 2017). It is one of the world's most important causes of disability-adjusted life years (DALYs) lost and the second global cause of years lived with disability (YLDs) (Hay et al. 2025).

Both early diagnosis and the initiation of effective antidepressant treatment are key to controlling symptoms, improving functioning and avoiding complications. However, there is difficulty in detecting depression from the patient's first contacts with the healthcare system, with diagnostic sensitivity in primary care estimated at around 50%, meaning that approximately half of cases are not detected (Mitchell, Vaze, and Rao 2009), and continued follow-up is required (often for too long) before diagnosis, which may lead to disease progression and avoidable medical complications.

Voice analysis could be a useful aid in the diagnosis of depression.

The possible clinical usefulness in depression of quantitative analysis of certain vocal speech characteristics was demonstrated about 50 years ago (Szabadi, Bradshaw, and Besson 1976), and the development of Artificial Intelligence and Machine Learning techniques has facilitated research in this area over the last 15 years (Almaghrabi, Clark, and Baumert 2023; Briganti and Lechien 2025). Different machine-learning models for acoustic voice analysis have achieved sensitivities and specificities above 70%, although with great variability in efficiency relative to sample size, ranging from the use of very brief speech fragments (as brief as 25 s) in very large non-clinical populations (more than 14,000 subjects) (Mazur et al. 2025) to longer fragments (between 7 and 50 min) in smaller samples of patients diagnosed with depression (usually between 22 and 158 patients) (Lu et al. 2025), including contexts at risk of isolation and unwanted loneliness as occurs in older populations (Martinez-Nicolas et al. 2025).

All these findings, although potentially applicable to the design of more homogeneous pilot studies, produce evidence that still suffers from selection biases, predominantly cross-sectional designs and shortcomings in external validation, which currently prevents translation into routine clinical practice (Briganti and Lechien 2025).

In summary, although preliminary evidence suggests that voice analysis may be considered a potential digital biomarker with complementary clinical utility in the diagnosis of depression, there is currently insufficient evidence to support its routine use, as further validation in diverse populations and standardization of speech data acquisition, processing and analysis are required (Almaghrabi, Clark, and Baumert 2023), in addition to longitudinal follow-up designs beyond cross-sectional associations.

This protocol addresses the methodological shortcomings mentioned above, proposing a prospective longitudinal framework in a clinical setting that combines diagnostic specificity (a population of patients diagnosed with moderate or severe depression) with breadth of presentations (both inpatient and outpatient consultation) and treatments (including patients receiving any type of psychotherapeutic, pharmacological or neurostimulation treatment, or even patients who decide not to receive treatment but remain under watchful follow-up), for the collection and analysis of speech data in Spanish-speaking patients with major depressive disorder and healthy controls. By integrating acoustic, paralinguistic and linguistic features and anchoring them to validated physician-administered clinical scales, this protocol seeks to advance research on diagnosis and follow-up of depression assisted by voice analysis, moving from exploratory modeling toward a clinically relevant and reproducible methodology. This is an exploratory pilot study of methodological validity in the context of an industrial doctorate.

# 8. RESEARCH HYPOTHESIS AND OBJECTIVES HYPOTHESIS

Major depressive disorder and its clinical course are associated with changes in certain acoustic, paralinguistic and linguistic characteristics of voice and speech that can be analyzed in a standardized and reproducible way in routine clinical practice.

**PRIMARY AND SECONDARY OBJECTIVES.**

The overall objective of this study is to develop and formalize a standardized, reproducible and clinically grounded protocol for the detection and longitudinal monitoring of major depressive disorder using speech-derived digital biomarkers, with a design that addresses the main limitations of current evidence: methodological heterogeneity, scarcity of longitudinal data and underrepresentation of non-English-speaking clinical populations.

Primary objective:

The primary objective is to establish a reproducible clinical framework for extracting and analyzing acoustic, paralinguistic and linguistic features from semi-structured clinical interviews, enabling discrimination between patients with a medical diagnosis of major depressive disorder and healthy controls, using voice-based models anchored to validated clinical reference standards (validated symptom-intensity scores objectified by the physician).

Secondary objectives:

1. To build a longitudinal Spanish-language clinical speech corpus comprising repeated voicerecordings from patients with major depressive disorder and single-session recordings from healthy controls.
2. To characterize the relationship between speech-derived digital biomarkers andphysician-rated measures of depression severity, including their evolution over time.
3. To assess the added value of multimodal approaches combining acoustic, paralinguistic andlinguistic features compared with unimodal models.
4. To evaluate intra-individual vocal changes across multiple assessment time points and theirassociation with changes in clinical severity.
5. To provide a transparent methodological framework that may facilitate future replication,cross-comparison and external validation in other clinical presentations and other diagnoses, languages and sociocultural settings.
6. To explore whether any treatment modality is more strongly associated with voice changesand clinical evolution.

# 9. RESEARCH METHODS

**a) Study design:**

This is an observational, prospective longitudinal study with a control group conducted in both outpatient and hospital clinical settings. The total planned duration of the study is 24 months. Two types of participants will be included: (1) a prospective cohort of patients with a medical diagnosis of major depressive disorder (MDD) and (2) healthy control participants with no history of psychiatric disorders. Patients with depression will receive 5 repeated monthly assessments over 4 months, while control participants will be assessed once under identical recording conditions.

The data will be obtained by reviewing medical records in the CUN information system. The study investigators will ensure the confidentiality of subject data and will ensure compliance at all times with Law 15/1999 on the protection of personal data and RD 1720/2007.

**b) Setting:**

Broad inclusion and exclusion criteria have been chosen to allow the inclusion of patients representative of real clinical practice. The study population consists of patients with MDD followed in the Department of Psychiatry of Clinica Universidad de Navarra, both in outpatient consultation and inpatient care, regardless of their treatment (psychotherapeutic, pharmacological or neurostimulation, or even patients who decide not to receive treatment but remain under watchful follow-up), with the following inclusion criteria and no exclusion criteria:

# Inclusion/exclusion criteria for patients

# Inclusion

1. Patients aged 18 years or older,
2. Spanish-speaking (native speakers or with sufficient fluency to conduct a clinical interview)
3. Able to understand the study procedures and provide written informed consent, in the investigator's judgment.
4. With a medical diagnosis of moderate or severe unipolar depression (with or without psychotic symptoms) according to ICD-10 diagnostic categories (Moderate depressive episode (F32.1) or severe depressive episode (F32.2 and F32.3), Recurrent depressive disorder, current episode moderate (F33.1) or severe (F33.2 and F33.3)), regardless of their treatment (psychotherapeutic, pharmacological or neurostimulation, or decision not to receive treatment).

# Exclusion

1. Severe neurological disorders affecting speech production or cognitive function (such assevere dementia). Non-severe neurological disorders or non-severe dementias are allowed.
2. Severe voice or speech disorders unrelated to depression that prevent the clinical interviewfrom being conducted through verbal language.
3. State of intoxication due to substance use at the time of assessment.
4. Other serious medical conditions that interfere with participation in this study or withspeech production.

# Inclusion/exclusion criteria for controls

# Inclusion

1. Age 18 years or older,

Exclusion

1. Personal history of depression or any other psychiatric disorder.
2. Severe neurological disorders affecting speech production or cognitive function (such assevere dementia). Non-severe neurological disorders or non-severe dementias are allowed.
3. Severe voice or speech disorders unrelated to depression that prevent the clinical interviewfrom being conducted through verbal language.
4. State of intoxication due to substance use at the time of assessment.
5. Other serious medical conditions that interfere with participation in this study or withspeech production.

**c) Participant selection, Visits and Variables:**

# Participant selection

Patients will be invited by the physicians of the research team in their routine clinical practice and recruited consecutively according to eligibility criteria. Controls will be invited by members of the research team and, if they agree, will be referred to one of the physicians on the research team to undergo the V0 visit. As this is an observational study, no randomization or blinding procedures apply. All participants will undergo identical recording and assessment procedures within their respective cohorts.

# Visit schedule

The study will be conducted according to a structured sequence of clinical visits designed to ensure standardized data collection while minimizing participant burden. All study procedures will be performed by qualified medical staff in an outpatient consultation or inpatient setting.

**Recruitment, consent and initial assessment visit (baseline visit, V0).** Potential participants will be informed about the objectives, scope and methodology of the study on depression detection through voice analysis and voice transcription. The non-invasive nature of the procedure, consisting of audio-recording a specific part of the medical interview, the confidentiality of the data collected and the exclusive use of the data for research purposes will be explicitly explained. Participants who express interest in participating in the study will be provided with the Participant Information Sheet and the informed consent form, and will be given sufficient time to read the documentation and ask questions.

Written informed consent will be obtained before initiating any study-related procedure. A structured clinical interview will then be conducted to collect basic sociodemographic and clinical information, followed by the initial psychometric assessment using the clinical scales defined in the protocol. During the same session, voice recordings will be obtained under controlled conditions following a standardized protocol that includes a combination of structured and open-ended questions. These recordings will form the basis for subsequent extraction of acoustic, paralinguistic and linguistic variables.

Healthy controls will only undergo the V0 visit. Only patients will receive follow-up visits.

**Follow-up visits (Visits V1, V2, V3 and V4).** Patients with major depressive disorder will be invited to attend four follow-up visits, coinciding with scheduled medical visits as part of routine clinical follow-up, approximately every 4 weeks, with an allowed window of +/-7 days. At each follow-up visit, voice and speech recordings will be repeated using the same standardized protocol applied at baseline, allowing longitudinal assessment of intra-individual changes in speech-derived variables. In addition, measures of depression severity, both physician-objectified and patient self-reported, will be updated, and any relevant clinical change, including treatment adjustments or adverse events, will be documented.

The data collected at follow-up visits will allow analysis of the temporal trajectories of acoustic, paralinguistic and speech characteristics in relation to changes in depressive symptomatology, supporting both cross-sectional and longitudinal modeling approaches.

**Exposure variables:**

- Medical diagnosis of moderate or severe unipolar depression (with or without psychoticsymptoms) according to ICD-10 diagnostic categories (Moderate depressive episode (F32.1) or severe depressive episode (F32.2 and F32.3), Recurrent depressive disorder, current episode moderate (F33.1) or severe (F33.2 and F33.3)), regardless of treatment (psychotherapeutic, pharmacological or neurostimulation, or decision not to receive treatment).

**Primary outcomes:**

- Voice recording protocol and variables from multimodal analysis of voice and speech (V0, V1 at 30 (+/-7) days, V2 at 60 (+/-7) days, V3 at 90 (+/-7) days and V4 at 120 (+/-7) days).

# Voice recording protocol

Only the part of the medical interview corresponding to the MADRS scale (between 10 and 30 min), which will include an initial free-speech section, will be recorded. Voice recordings will be obtained using a Jabra Speak 510 USB omnidirectional microphone. The microphone will be placed at a distance of no more than 20 cm from the participant's mouth. Recordings will be made in a quiet hospital environment (consultation room). By intentionally avoiding a high-end studio microphone, the aim is to maximize ecological validity and facilitate possible applicability in routine clinical practice. This approach is consistent with previous studies in the field, which have used non-specialized recording devices (Menne et al. 2024; Wasserzug et al. 2023).

Audio recordings will be stored in uncompressed WAV format to preserve the signal. All files will be labeled with pseudonymized alphanumeric codes. During preprocessing, the interviewer's speech will be removed and only the participant's speech signal will be used for analysis.

Variables from the multimodal analysis of voice and speech. The variables included in this study have been selected from evidence from previous studies:

1. Acoustic:
   1.1. Pitch (mean F0, standard deviation, range): Frequency in Hz.
   1.2. Energy / intensity (mean, standard deviation): dB.
   1.3. MFCCs (coefficients 1-13, mean / standard deviation): Cepstral coefficients.
   1.4. Harmonics-to-noise ratio (HNR) in dB.
   1.5. Jitter (%).
   1.6. Shimmer (%).
   1.7. Frequency-based spectral descriptors with spectral centroid / roll-off / slope (Hz ornormalized units).
   1.8. Formants (mean / bandwidth F1-F3), frequency (Hz) and bandwidth (Hz).
   1.9. Spectral entropy / prosodic entropy: entropy index (dimensionless).
   1.10. Embedding-based acoustic features (for example, Wav2Vec2, HuBERT) (high-dimensionalvector representations).

# 2. Paralinguistic

2.1. Speech rate / articulation rate / pause duration: Words per second (w/s); pause duration in seconds (s).
2.2. Prosodic range (F0 and intensity variability): Range (Hz, dB) or coefficient of variation (%).
2.3. Pause ratio (silence / speech): Proportion (0-1) or percentage (%).

# 3. Linguistic

3.1. Sentiment polarity (mean per utterance): Continuous scale from -1 (negative) to +1 (positive).
3.2. Negative emotional word proportion: Percentage (%) of words with negative valence.
3.3. First-person singular pronouns: Percentage (%) or frequency per 100 words.
3.4. Lexical diversity (MTLD, Honore, TTR): Lexical diversity indices (dimensionless).
3.5. Word count / mean sentence length: Number of words; words per sentence.
3.6. Lexical sentiment variance (intrasentence): standard deviation of sentiment scores (-1 to +1).
3.7. Lexical coherence / cohesion (BERT embedding similarity): Cosine similarity score (0-1).
- Intensity of depressive symptoms objectified by the physician (V0, V1 at 30 (+/-7) days, V2 at60 (+/-7) days, V3 at 90 (+/-7) days and V4 at 120 (+/-7) days), using the Montgomery-Asberg Depression Rating Scale (MADRS) (Montgomery and Asberg 1979; Lobo et al. 2002) (clinical scale completed by the physician).
- Global clinical severity objectified by the physician (V0, V1 at 30 (+/-7) days, V2 at 60 (+/-7) days, V3 at 90 (+/-7) days and V4 at 120 (+/-7) days), using the Clinical Global Impression scale (CGI) (Guy 1976) (clinical scale completed by the physician).

**Secondary outcomes:**

- Main diagnosis (baseline): type of unipolar major depressive disorder.
- Severity of depressive symptoms reported by the patient (V0, V1 at 30 (+/-7) days, V2 at 60 (+/-7) days, V3 at 90 (+/-7) days and V4 at 120 (+/-7) days), using the Patient Health Questionnaire - 9 (PHQ-9) (self-administered questionnaire).
- Psychiatric comorbidities (including history of self-harm and suicidal ideation and behavior,duration of the current episode, time since initial diagnosis and number of previous episodes) and general medical comorbidities, with concomitant medications and, if available, CYP or pharmacodynamic isoforms (baseline).
- Anthropometric, demographic and lifestyle variables (baseline): sex, age, marital status, placeof residence, birth order among siblings, height, weight, BMI, physical exercise practice (intensity, frequency), diet (healthy/unhealthy), level of school, professional or academic training, family care activity (care of children or dependent relatives), employment activity (yes/no, type), social relationships, religious belief (yes/no), community religious practice (frequency).
- Pharmacological treatment variables (V0, V1 at 30 (+/-7) days, V2 at 60 (+/-7) days, V3 at 90 (+/-7) days and V4 at 120 (+/-7) days): dose, dosing interval, time of administration, duration of treatment (for each drug received by the patient).
- Non-pharmacological treatment variables (V0, V1 at 30 (+/-7) days, V2 at 60 (+/-7) days, V3 at 90 (+/-7) days and V4 at 120 (+/-7) days): lifestyle hygienic-dietary measures (sleep
- hygiene, healthy diet pattern, regular physical exercise, cessation of tobacco and/or toxic substances). Type of psychotherapy, number/frequency of psychotherapy sessions, repetitive transcranial magnetic stimulation (type and frequency); electroconvulsive therapy (type and frequency).
- Other variables (baseline): family psychiatric history, current and previousstressful-precipitating/traumatic life events, toxic habits and type of toxic substances consumed.

d) Data sources:

Information on the variables for each participant will be collected at each visit in a pseudonymized case report form.

**e) Sample size**

The study is planned with a total sample of 75 subjects, composed of 25 patients with depression and 50 healthy controls, which constitutes a clinically realistic design consistent with the available recruitment capacity.

The sample has an unbalanced distribution between groups, a circumstance that has been taken into account in the methodological approach and in the interpretation of results. This configuration allows the study to be approached from a dual perspective. On the one hand, it enables comparison between patients with depression and healthy controls in order to explore the existence of vocal differences associated with the disorder. On the other hand, the design incorporates a longitudinal component in the clinical group, which allows analysis of intra-individual evolution throughout follow-up and assessment of the potential of voice not only as a detection tool but also as a monitoring tool. Thus, although the sample is not symmetrical between groups, its structure enables complementary information to be extracted and maximizes the scientific value of the available data within a realistic clinical context. Although the number of patients with depression is limited, the added value of the study lies in its longitudinal component, since these 25 patients will be assessed at 5 visits (baseline and 4 follow-up visits), generating 125 records in the clinical group and allowing not only comparison between patients and controls but also analysis of the intra-individual evolution of vocal biomarkers in relation to the clinical course.

However, because the 5 observations from each patient with depression are not independent of one another but are correlated, the raw number of records cannot be interpreted as the effective sample size. To correct this, the design effect associated with repeated measures is considered, calculated using the expression DEFF=1+(m-1)ICC, where m is the number of measurements per subject and ICC is the assumed intraclass correlation coefficient between visits. In this case, taking m=5 and assuming a moderate correlation of ICC=0.2 gives: DEFF=1+(5-1)*0.2=1+0.8=1.8. Therefore, the effective size of the depression group can be approximated as nef=125/1.8=69.4. Adding this value to the 50 independent controls, the total effective sample size of the study would be approximately 69.4+50=119.4 effective observations.

**f) Data management.**

With regard to clinical data, only the persons designated by the sponsor, the monitor, the investigator and his/her team of collaborators, and the relevant health authorities will have access to the medical record data and case report forms necessary to achieve the project objectives.

Regarding pseudonymized voice recordings, they will be transferred in full and without distortion, physically from an external memory device to NNBi servers, where they will be stored until 31/12/2029, and destroyed by that date at the latest once this project has been completed. NNBi undertakes to destroy all recordings and their backup copies before 01/01/2030. The NNBi servers intended for storage of anonymized audios guarantee the security, confidentiality, integrity and availability of the data in accordance with current regulations. NNBi is certified under the National Security Scheme (ENS) at High level and under ISO 27001, which accredits a security framework aligned with international best practices. In this context, advanced technical and organizational measures are applied: encryption of information at rest and in transit, strict role-based access control and the principle of least privilege, robust authentication, segregation of environments, and classification of information according to its sensitivity. In addition, monitoring and logging of accesses and operations are ensured in order to detect improper uses, together with integrity protection mechanisms and prevention of unauthorized alterations. Finally, information life-cycle management includes encrypted backups, incident recovery procedures and secure deletion at the end of the defined retention period, minimizing risk to patients and ensuring responsible data processing.

The sponsor and study investigators must guarantee the confidentiality of subject data and ensure compliance at all times with Organic Law 3/2018 on the protection of personal data and Regulation (EU) 2016/679 of the European Parliament and of the Council of 27 April 2016 on Data Protection.

**g) Data analysis.**

It comprises the following statistical analyses and modeling strategy:

An initial descriptive analysis of all extracted variables will be performed. For quantitative variables (acoustic, paralinguistic and linguistic), the mean and standard deviation will be calculated, while percentages and frequencies will be used for qualitative variables.

To fulfill the study objectives, the analysis will be structured around three axes:

1. Association and Diagnostic Discrimination: Correlation coefficients (Pearson/Spearman) will be used to assess the association between continuous biomarkers and symptom-intensity scales (MADRS, PHQ-9 and CGI). Classification models will be used for discrimination between patients and healthy controls. The Area Under the Curve (AUC) will be reported as the main performance metric, together with the Odds Ratio and its 95% confidence intervals.
2. Multimodal Assessment: A systematic comparison will be performed between unimodal models (based exclusively on audio or text) and multimodal fusion models. The use of advanced language models is contemplated for processing transcriptions and extracting complex semantic features. This analysis will quantify the added value of combining acoustic, paralinguistic and linguistic features in predicting clinical severity.
3. Longitudinal and Intra-individual Analysis: Mixed-effects models or finite-difference models will be used to assess the evolution of biomarkers over time and their relationship with change in clinical severity. These models allow management of intra-subject correlation due to repeated measures and control of inter-individual variability.

Validation and Transparency: All assessments will be performed using a participant-level data-splitting strategy (Leave-One-Group-Out), ensuring that all visits from the same subject belong exclusively to either the training group or the test group in order to avoid overfitting bias. This aspect is methodologically relevant because of the need to use one data set for model training and another data set to validate the results. Both groups will include patients and healthy controls.

To ensure clinical interpretation of the results, feature contribution analyses will be performed (attribution based on Shapley values or SHAP), enabling identification of which specific signals (e.g., pauses, pitch variations or lexical content) drive the model predictions. Analyses will be performed using specialized statistical software (Python v3.x). P values < 0.05 will be considered statistically significant.

**h) Quality control.**

Control of the data collected in the CRF will be ensured by the research team itself. The principal investigator will monitor compliance with the study protocol.

**i) Limitations of the research methods.**

The following limitations and anticipated challenges of the proposed protocol must be recognized, especially those inherent to voice-based clinical studies that seek to balance standardization, ecological validity and translational feasibility:

1) First, the clinical scales included (MADRS, CGI and PHQ-9) are used as the main reference standards for symptom intensity and severity, but they are subject to measurement variability and lack diagnostic validity. It is very important to emphasize that the current protocol does not seek to replace diagnostic clinical judgment, but rather to complement it by identifying speech-derived markers that correlate with established measures of depressive severity.
2) Although speech recordings are obtained under controlled clinical setting conditions (consultation room, hospital ward), significant inter-individual variability is expected in speaking style, emotional expressiveness and engagement during clinical interviews. To balance standardization with real-world clinical conditions, a semi-structured physician-administered interview protocol is used, which includes a space for the patient's free and spontaneous speech in order to elicit naturalistic speech. This improves ecological validity and clinical relevance, but may introduce variability in speech content, duration and topic coverage, which in turn may affect both acoustic and linguistic measurements.
3) Longitudinal data collection presents practical challenges. Repeated assessments increaseparticipant burden and may lead to missed visits or incomplete follow-up data. To mitigate this risk, follow-up visits are scheduled within flexible time windows and all procedures are designed to be brief, highly adapted to routine clinical practice and with minimal added burden for the patient. Nevertheless, a certain degree of attrition is expected and will be addressed explicitly in the analytical strategy.
4) The planned sample size remains moderate. Although the longitudinal structure providesvaluable information on intra-individual vocal trajectories, a moderate sample may limit statistical power, increase uncertainty in performance estimates,

restrict exploration of complex interaction effects and increase the risk of overfitting, especially in higher-dimensional or multimodal models.

5) Technical variability in recording conditions, such as background noise or microphoneposition, may influence acoustic measurements. The protocol addresses this by using a standardized recording device and placement, intentionally avoiding high-level or laboratory equipment to maximize clinical applicability. This compromise prioritizes real-world applicability over maximum signal fidelity and may reduce the signal-to-noise ratio for certain acoustic biomarkers.
6) The study is conducted in a single clinical center and exclusively in Spanish-speakingparticipants, which may restrict external validity in healthcare systems serving populations with dialects or other linguistic contexts. However, this approach also addresses a relevant gap in the literature, which has predominantly focused on English-language data sets.
7) Finally, the exclusion of certain highly complex features, such as the mandatory use of acoustic embeddings, may limit maximum predictive performance. However, this decision is consistent with the study's emphasis on methodological reproducibility, interpretability and clinical deployment, and allows the main feature set to remain transparent, auditable and feasible to calculate in routine hospital settings.

# 10. PROTECTION OF PERSONS SUBJECT TO THE STUDY

a) **Benefit-risk assessment for research subjects, if applicable.**

Not applicable, as this study analyzes variables collected in the medical record as part of routine clinical practice, clinical questionnaires specified in the variables section, and consent for recording part of the medical interview.

b) **Considerations on information to subjects and informed consent.**

Once the study has been fully explained to the patient, written informed consent will be obtained from the patient or from his/her guardian or legal representative before participation in the study becomes effective.

The investigator (or delegated person) will also sign and date the consent form. The investigator will file the original signed Consent Form in the center's Investigator File. The patient will receive the Informed Consent Form and will be informed that participation in the study is voluntary and that he/she may withdraw at any time without prejudice to subsequent medical care. Neither the Patient Information Sheet nor the Informed Consent may be modified without the agreement of the Research Ethics Committee with medicines and the sponsor.

A copy of the Informed Consent Form must be held by the patient.

The Consent Form includes information regarding the need to access Medical Records.

**c) Data confidentiality:**

In order to guarantee data confidentiality, study patients will be identified with a number, assigned sequentially according to the order of inclusion. The case report forms (CRF), reports and study communications will be identified with that number. Only the sponsor, the investigator and his/her team of collaborators, the Research Ethics Committee with medicines that supervises the study, and the relevant health authorities will have access to the identified material necessary to achieve the objectives of the study, according to the patient's authorization.

The contents of the CRFs as well as the documents generated during the study will be protected against unauthorized use by persons outside the study and will therefore be considered strictly confidential and will not be disclosed to third parties except those specified in the previous paragraph.

The sponsor and study investigators must guarantee the confidentiality of subject data and ensure compliance at all times with Organic Law 3/2018 on the protection of personal data and European Regulation (EU) 2016/679.

# d) Interference with the physician's prescribing habits

The conduct of this prospective study does not modify medical prescribing habits, since patients are included regardless of their treatment (psychotherapeutic, pharmacological or neurostimulation), including patients who decide not to receive treatment but remain under watchful follow-up. As a secondary objective, the study will explore whether any treatment modality is more strongly associated with voice changes and clinical evolution.

# 11. MANAGEMENT AND REPORTING OF ADVERSE REACTIONS and other relevant events occurring during the study

Cases of suspected serious and unexpected adverse reactions will be communicated through the official channel established at CUN.

# DEFINITIONS

An adverse event is defined as any health-damaging occurrence in a patient or subject in a clinical study treated with a drug, even if it does not necessarily have a causal relationship with that treatment.

An AE may therefore be any unfavorable and unintended sign (including an abnormal laboratory finding), symptom or disease temporally associated with the use of an investigational medicinal product, whether or not related to the investigational medicinal product.

A serious adverse event is defined as any adverse event or adverse reaction that, at any dose:

- Results in the death of the patient.
- Threatens the life of the patient (in the investigator's opinion, the patient at the time of the AEor AR is at actual risk of death; this does not refer to the AE/AR hypothetically being able to have caused death had it been more intense).
- Requires hospitalization or prolongation of the patient's hospitalization
- Causes permanent or significant disability or incapacity
- Leads to a congenital anomaly or malformation

An adverse reaction is considered any harmful and unintended reaction to an investigational medicinal product, regardless of the dose administered. Unlike an AE, in the case of an adverse reaction there is a suspected causal relationship between the investigational medicinal product and the adverse event.

Determination of the possible relationship with study treatment must be made according to the following definitions:

- NOT RELATED: There is no evidence of any causal relationship.
- UNLIKELY: There is little evidence to suggest a causal relationship (e.g., the event did notoccur within a reasonable period after administration of the study drug/procedure). There is another reasonable explanation for the event (e.g., the patient's clinical condition, other concomitant treatments).
- POSSIBLE: There is evidence suggesting a possible causal relationship (e.g., because the eventoccurred within a reasonable time after administration of the study drug). However, the influence of other factors may have contributed to the event (e.g., the patient's clinical condition, other concomitant treatments).
- PROBABLE: There is evidence suggesting a causal relationship and the influence of otherfactors is unlikely.
- DEFINITE: There is clear evidence suggesting a causal relationship and a possiblecontribution from other factors can be ruled out.

Serious Unexpected Adverse Reaction (SUSAR). Any serious adverse reaction whose nature, severity or consequences do not correspond to the reference information for the medicinal product.

The unexpected nature of an adverse reaction is based on the fact that it has not been previously observed and will not be based on what could be anticipated according to the pharmacological properties of the medicinal product.

REPORTING OF ADVERSE EVENTS.

Cases of serious and unexpected adverse reactions must be systematically recorded by the healthcare professional and transmitted to the authorities through the official channel established for this purpose at CUN. This study does not modify the therapeutic regimen of the participants, which includes the obligations inherent to the administration of medicines and the detection and reporting thereof to pharmacovigilance systems when appropriate. The healthcare professional responsible for treatment will be in charge of reporting suspected adverse reactions when appropriate through the usual channel established for this purpose at CUN.

# 12. WORK PLAN

**(tasks, milestones and study chronology).**

| **Activity** | **Start date** | **End date** |
| --- | --- | --- |
| Data collection | May 2026 | December 2027 |
| Analysis and interpretation of the data obtained | January 2028 | February 2028 |
| Writing | March 2028 | June 2028 |
| Final results report | September 2028 |  |

13. **PLANS FOR DISSEMINATION AND COMMUNICATION OF STUDY RESULTS.**

The results will be submitted for publication in specialized peer-reviewed scientific journals.

14. **REFERENCES.**

Akiskal, Hagop S. 2017. "13.4 Mood Disorders: Clinical Features." In Kaplan and Sadock's Comprehensive Textbook of Psychiatry, Tenth Edition, edited by BJ Sadock, VA Sadock, and P Ruiz, 1630-60.

Almaghrabi, Shaykhah A., Scott R. Clark, and Mathias Baumert. 2023. "Bio-Acoustic Features of Depression: A Review." Biomedical Signal Processing and Control 85 (August):105020. https://doi.org/10.1016/J.BSPC.2023.105020.

American Psychiatric Association. 2013. "Diagnostic and Statistical Manual of Mental Disorders (5th Ed.) (DSM-5)."

Arias-de la Torre, Jorge, Gemma Vilagut, Amy Ronaldson, Ioannis Bakolis, Alex Dregan, Vicente Martin, Gonzalo Martinez-Ales, et al. 2023. "Prevalence and Variability of Depressive Symptoms in Europe: Update Using Representative Data from the Second and Third Waves of the European Health Interview Survey (EHIS-2 and EHIS-3)." The Lancet Public Health 8 (11): e889-98. https://doi.org/10.1016/S2468-2667(23)00220-7.

Briganti, Giovanni, and Jerome R. Lechien. 2025. "Speech and Voice Quality as Digital Biomarkers in Depression: A Systematic Review." Journal of Voice. https://doi.org/10.1016/j.jvoice.2025.05.002.

Garcia-Portilla, M.P., M.T. Fernandez-Bascaran, P.A. Saiz-Martinez, M.T. Bobes-Bascaran, M. Bousono-Garcia, and J. Bobes-Garcia. 2022. Banco de Instrumentos Basicos Para La Practica de La Psiquiatria Clinica. Octava ed.

Guy, W. 1976. Early Clinical Drug Evaluation (ECDEU) Assessment Manual. Rockville, Maryland: National Institute of Mental Health.

Hay, Simon I., Kanyin Liane Ong, Damian F. Santomauro, A. Bhoomadevi, Mohammad Amin Aalipour, Hasan Aalruz, Hazim S. Ababneh, et al. 2025. "Burden of 375 Diseases and Injuries, Risk-Attributable Burden of 88 Risk Factors, and Healthy Life Expectancy in 204 Countries and Territories, Including 660 Subnational Locations, 1990-2023: A Systematic Analysis for the Global Burden of Disease Study 2...." The Lancet 406 (10513): 1873-1922. https://doi.org/10.1016/S0140-6736(25)01637-X.

Lobo, Antonio, Lorenzo Chamorro, Antonio Luque, Rafael Dal-Re, Xavier Badia, Eva Baro, Concepcion Lacamara, et al. 2002. "[Validation of the Spanish Versions of the Montgomery-Asberg Depression an Hamilton Anxiety Rating Scales]." Medicina Clinica 118 (13): 493-99. https://doi.org/10.1016/S0025-7753(02)72429-9.

Lu, Wei, Xiaowei Tang, Chuan Huang, Man Wei, Chengxin Bai, Xuqing Fan, and Dongmei Wu. 2025. "Diagnostic Accuracy of Traditional and Deep Learning Methods for Detecting Depression Based on Speech Features: A Systematic Review and Meta-Analysis." BMC Psychiatry 25 (1). https://doi.org/10.1186/S12888-025-07628-Z.

Martinez-Nicolas, Israel, Diego Criado, Fernando Gordillo, Francisco Martinez-Sanchez, and Juan Jose G. Meilan. 2025. "Speech Analysis for Detecting Depression in Older Adults: A Systematic Review." Frontiers in Psychology 16. https://doi.org/10.3389/FPSYG.2025.1715538.

Mazur, Alexa, Harrison Costantino, Prentice Tom, Michael P. Wilson, and Ronald G. Thompson. 2025. "Evaluation of an AI-Based Voice Biomarker Tool to Detect Signals Consistent With Moderate to Severe Depression." Annals of Family Medicine 23 (1): 60-65. <https://doi.org/10.1370/AFM.240091>.

Menne, Felix, Felix Dorr, Julia Schrader, Johannes Troger, Ute Habel, Alexandra Konig, and Lisa Wagels. 2024. "The Voice of Depression: Speech Features as Biomarkers for Major Depressive Disorder." BMC Psychiatry 24 (1): 794. https://doi.org/10.1186/s12888-024-06253-6.

Merikangas, KR, and Z Rihmer. 2017. "13.2 Mood Disorders: Epidemiology." In Kaplan and Sadock's Comprehensive Textbook of Psychiatry, Tenth Edition, 1614-19.

Mitchell, Alex J., Amol Vaze, and Sanjay Rao. 2009. "Clinical Diagnosis of Depression in Primary Care: A Meta-Analysis." The Lancet 374 (9690): 609-19. <https://doi.org/10.1016/S0140-6736(09)60879-5>.

Montgomery, S. A., and M. Asberg. 1979. "A New Depression Scale Designed to Be Sensitive to Change." British Journal of Psychiatry 134 (4): 382-89. https://doi.org/10.1192/BJP.134.4.382,.

Szabadi, E., C. M. Bradshaw, and J. A.O. Besson. 1976. "Elongation of Pause-Time in Speech: A Simple, Objective Measure of Motor Retardation in Depression." The British Journal of Psychiatry 129 (6): 592-97. https://doi.org/10.1192/BJP.129.6.592.

Wasserzug, Yael, Yoav Degani, Mili Bar-Shaked, Milana Binyamin, Amit Klein, Shani Hershko, and Yechiel Levkovitch. 2023. "Development and Validation of a Machine Learning-Based Vocal Predictive Model for Major Depressive Disorder." Journal of Affective Disorders 325 (March):627-32. https://doi.org/10.1016/j.jad.2022.12.117.

World Health Organization. 2016. "ICD-10 : International Statistical Classification of Diseases and Related Health Problems : Tenth Revision, 2nd Ed."
