# Supplementary material for: The VOICE-DEP study protocol: Multimodal analysis of voice and speech during medical interviews to support diagnosis and longitudinal monitoring of major depressive disorder
Source: PLoS One. 2026 Sep 10;21(9):e0353827. doi: 10.1371/journal.pone.0353827 (PMC13561378; doi:10.1371/journal.pone.0353827)
Supplement: Supplementary file 2 [file pone.0353827.s001.doc]

# PROTOCOLO

**1.	TÍTULO DESCRIPTIVO Y VERSIÓN DEL PROTOCOLO.**

ANÁLISIS MULTIMODAL DE LA VOZ Y DEL DISCURSO DURANTE LA ENTREVISTA MÉDICA COMO HERRAMIENTA DE APOYO AL DIAGNÓSTICO Y EVALUACIÓN DE LA RESPUESTA AL TRATAMIENTO EN EL TRASTORNO DEPRESIVO.

**Título corto**: Estudio VOICE-DEP: evaluación de la depresión asistida por análisis de la voz.

Versión 2, de fecha 27 de abril de 2026.

**2.	PROMOTORES DEL ESTUDIO:**

CLINICA UNIVERSIDAD DE NAVARRA

Avenida Pío XII, 36

31008 Pamplona

NNBi 2020 S.L

Pol. Noain Esquiroz · calle H nº2

of. p1 12-13 · 31191 Esquiroz, Navarra

Empresa especializada en análisis e interpretación de datos en salud, que desarrolla soluciones personalizadas de inteligencia artificial y modelos predictivos (Machine Learning) aplicados principalmente a datos clínicos.

**3.	EQUIPO INVESTIGADOR**

Investigador Principal: Patricio Molero Santos. Doctor en Medicina. Especialista en Psiquiatría. Clínica Universidad de Navarra.

Azucena Aldaz Pastor. Doctora en Farmacia. Especialista en Farmacia Hospitalaria. Clínica Universidad de Navarra.

Maialen Zabalza Zudaire, Graduada en Matemáticas y Máster Universitario en Inteligencia Artificial. Gestión Técnica y Especialista en I+D. NNBi 2020 S.L.

Onintza Sayar Beristain, Doctora en Farmacia, promotora y Gerente de la empresa NNBi 2020 S.L.

Felipe Ortuño Sánchez Pedreño. Doctor en Medicina. Especialista en Psiquiatría. Clínica Universidad de Navarra.

Paula Fructos Cordovilla. Médico Interno Residente en Psiquiatría. Clínica Universidad de Navarra.

Franco Fabricio Carpio Chanamé. Médico Interno Residente en Psiquiatría. Clínica Universidad de Navarra.

Esmeralda García Pérez. Médico Interno Residente en Psiquiatría. Clínica Universidad de Navarra.

Álvaro Ortiz Agudo. Médico Interno Residente en Psiquiatría. Clínica Universidad de Navarra.

Favio Emanuel Núñez Ortiz. Médico Interno Residente en Psiquiatría. Clínica Universidad de Navarra.

**4.	RESUMEN.**

a) Título: Análisis multimodal de la voz y del discurso durante la entrevista médica como herramienta de apoyo al diagnóstico y evaluación de la respuesta al tratamiento en el trastorno depresivo.

**Título corto**: Estudio VOICE-DEP: evaluación de la depresión asistida por análisis de la voz.

IP: Patricio Molero. Clínica Universidad de Navarra

b) Justificación y contexto.

El trastorno depresivo mayor (TDM) es una enfermedad grave, recurrente e incapacitante con una elevada prevalencia en nuestro medio, que se asocia a un incremento de morbimortalidad por riesgo de suicidio y otras causas tanto médicas generales como específicamente psiquiátricas asociadas, y supone una de las causas más importantes de años de vida perdidos ajustados por discapacidad. El diagnóstico precoz es un aspecto clave para el control de los síntomas, mejorar la funcionalidad y evitar complicaciones. Existe evidencia preliminar sugestiva de la consideración del análisis de la voz como un potencial biomarcador digital con utilidad clínica complementaria en el diagnóstico de la depresión. El objetivo general de este estudio es desarrollar y formalizar un protocolo estandarizado, reproducible y clínicamente fundamentado para la detección y monitorización longitudinal del trastorno depresivo mayor utilizando biomarcadores digitales derivados del habla, integrando características acústicas, paralingüísticas y lingüísticas ancladas a escalas clínicas validadas, para avanzar en la investigación del diagnóstico y seguimiento de la depresión asistido por el análisis de voz.

Se trata de un estudio piloto exploratorio de validez de metodología en el contexto de un doctorado industrial.

c) Hipótesis y objetivos de la investigación.

Hipótesis de trabajo:

El trastorno depresivo mayor y su evolución clínica se asocian a cambios en determinadas características de la voz y del discurso de tipo acústico, paralingüístico y lingüístico, que es posible analizar de un modo estandarizado y reproducible en un entorno de práctica clínica habitual.

Objetivo primario:

Establecer un marco clínico reproducible para la extracción y análisis de características acústicas, paralingüísticas y lingüísticas a partir de entrevistas clínicas semiestructuradas, permitiendo la discriminación entre pacientes con diagnóstico médico de trastorno depresivo mayor y controles sanos, utilizando modelos basados en la voz anclados en estándares de referencia clínicos validados (puntuaciones validadas de intensidad sintomática objetivados por el médico).

Objetivos secundarios:

1. Construcción de un corpus de habla clínica longitudinal en castellano, que comprenda grabaciones repetidas de voz de pacientes con trastorno depresivo mayor y grabaciones de una sola sesión de controles sanos.
2. Caracterizar la relación entre biomarcadores digitales derivados del habla y medidas de gravedad de la depresión valoradas por los médicos, incluyendo su evolución a lo largo del tiempo.
3. Evaluar el valor añadido de los enfoques multimodales que combinan características acústicas, paralingüísticas y lingüísticas en comparación con modelos unimodales.
4. Evaluar los cambios vocales intraindividuales a lo largo de múltiples puntos temporales de evaluación y su asociación con cambios en la gravedad clínica.
5. Proporcionar un marco metodológico transparente que pueda facilitar la replicación, la comparación cruzada y la validación externa futura en otras presentaciones clínicas y otros diagnósticos, lenguajes y entornos socioculturales.
6. Explorar si algún tipo de modalidad de tratamiento se asocia en mayor medida a los cambios en la voz con la evolución clínica.

d) Diseño del estudio.

Observacional, longitudinal prospectivo con grupo control.

e) Población.

Pacientes con TDM con seguimiento en el departamento de Psiquiatría de la Clínica Universidad de Navarra, tanto en consulta ambulatoria como en régimen de hospitalización, independientemente de su tratamiento (psicoterapéutico, farmacológico o de neuroestimulación, o incluso pacientes que deciden no recibir tratamiento, pero sí seguimiento con actitud expectante), con los siguientes criterios de inclusión y sin ningún criterio de exclusión:

Criterios de inclusión/exclusión para los pacientes

Inclusión

1. Pacientes con edad igual o superior a 18 años,
2. De habla castellana (nativos o con fluidez suficiente para mantener una entrevista clínica)
3. Con capacidad para comprender los procedimientos del estudio y proporcionar consentimiento informado por escrito, según el juicio del investigador.
4. Con diagnóstico médico de depresión unipolar moderada o grave (con o sin síntomas psicóticos) según las categorías diagnósticas de CIE-10 (Episodio depresivo moderado (F32.1) o grave (F32.2 y F32.3), Trastorno depresivo recurrente episodio actual moderado (F33.1) o grave (F33.2 y F33.3)), independientemente de su tratamiento (psicoterapéutico, farmacológico o de neuroestimulación, o decisión de no recibir tratamiento).

Exclusión

1. Trastornos neurológicos graves que afectan a la producción del habla o a la función cognitiva (como demencia severa). Se permiten trastornos neurológicos no graves o demencias no severas.
2. Trastornos graves de la voz o del habla no relacionados con la depresión que impidan la realización de la entrevista clínica a través del lenguaje verbal.
3. Estado de intoxicación por consumo de sustancias en el momento de la evaluación.
4. Otras condiciones médicas graves que interfieran con la participación en este estudio o la producción del habla.

Criterios de inclusión/exclusión para los controles

Inclusión

1.	Edad igual o superior a 18 años,

Exclusión

1.	Antecedentes personales de depresión o cualquier otro trastorno psiquiátrico.

2.	Trastornos neurológicos graves que afectan a la producción del habla o a la función cognitiva (como demencia severa). Se permiten trastornos neurológicos no graves o demencias no severas.

3.	Trastornos graves de la voz o del habla no relacionados con la depresión que impidan la realización de la entrevista clínica a través del lenguaje verbal.

4.	Estado de intoxicación por consumo de sustancias en el momento de la evaluación.

5.	Otras condiciones médicas graves que interfieran con la participación en este estudio o la producción del habla.

f) Variables.

-Diagnóstico médico de depresión unipolar moderada o grave (con o sin síntomas psicóticos) según las categorías diagnósticas de CIE-10 (Episodio depresivo moderado (F32.1) o grave (F32.2 y F32.3), Trastorno depresivo recurrente episodio actual moderado (F33.1) o grave (F33.2 y F33.3)), independientemente de su tratamiento (psicoterapéutico, farmacológico o de neuroestimulación, o decisión de no recibir tratamiento).

-Variables del análisis multimodal de la voz y del discurso (acústicas, paralinguísticas y lingüísticas, especificadas en el apartado de variables)

-Intensidad de síntomas depresivos objetivada por el médico, a través de la escala Montgomery-Asberg para la depresión (MADRS) (Montgomery and Asberg 1979; Lobo et al. 2002) (escala clínica realizada por el médico), tomada del Banco de instrumentos básicos para la práctica de la psiquiatría clínica (García-Portilla et al. 2022).

-Gravedad clínica global objetivada por el médico, a través de la escala de Impresión Clínica Global (ICG) (Guy 1976)(escala clínica realizada por el médico), tomada del Banco de instrumentos básicos para la práctica de la psiquiatría clínica (García-Portilla et al. 2022).

-Gravedad de los síntomas depresivos referida por el paciente, a través del Cuestionario de Salud del Paciente – 9 (PHQ-9) (cuestionario autoaplicado) tomado del Banco de instrumentos básicos para la práctica de la psiquiatría clínica (García-Portilla et al. 2022).

-Comorbilidades psiquiátricas y médicas generales (especificadas en el apartado de variables).

-Variables antropométricas, demográficas y hábitos de vida (especificadas en el apartado de variables).

-Variables de tratamiento farmacológico (especificadas en el apartado de variables).

-Variables de tratamiento no farmacológico (especificadas en el apartado de variables).

-Otras variables (especificadas en el apartado de variables).

g) Fuentes de los datos.

La información de las variables para cada participante se recogerá durante cada visita en un cuaderno de recogida de datos seudonimizado.

h) Tamaño de muestra del estudio.

La muestra prevista comprende un total de 175 grabaciones de voz. Se registrarán 25 pacientes con diagnóstico clínico de trastorno depresivo mayor en 5 puntos temporales (Vista 0 y 4 visitas mensuales) longitudinales, con un total de 125 registros longitudinales. Además, se registrarán 50 participantes sanos de control con un solo registro de voz basal (Visita 0). Justificación en el apartado de tamaño muestral.

i) Análisis de los datos.

Análisis estadísticos y estrategia de modelización especificados en el apartado de Análisis de datos.

j) Etapas y calendario.

| **Actividad** | **Fecha inicio** | **Fecha fin** |
| --- | --- | --- |
| Recogida de datos | mayo 2026 | diciembre 2027 |
| Análisis e interpretación de los datos obtenidos | enero 2028 | Febrero 2028 |
| Redacción | Marzo 2028 | Junio 2028 |
| Informe final de resultados | Septiembre 2028 |  |

**5.	MODIFICACIONES Y ACTUALIZACIONES**

Toda modificación sustancial del protocolo del estudio después del inicio de la recogida de los datos, con su justificación, la fecha y la indicación de la sección del protocolo que se ha visto afectada.

**6. ETAPAS**

Cuadro con el calendario previsto para, al menos, las siguientes etapas:

a) Inicio de la recogida de datos.

b) Final de la recogida de datos.

c) Informes de situación del estudio, si procede. (No procede).

d) Informes intermedios de los resultados del estudio, si procede. (No procede).

e) Informe final de los resultados del estudio.

| **Actividad** | **Fecha inicio** | **Fecha fin** |
| --- | --- | --- |
| Recogida de datos | mayo 2026 | diciembre 2027 |
| Análisis e interpretación de los datos obtenidos | enero 2028 | Febrero 2028 |
| Redacción | Marzo 2028 | Junio 2028 |
| Informe final de resultados | Septiembre 2028 |  |

**7.	JUSTIFICACIÓN Y CONTEXTO.**

La depresión (en este proyecto considerada según criterios diagnósticos de las categorías de episodios depresivos moderados o graves según la CIE-10 (World Health Organization 2016) y del trastorno depresivo mayor (TDM) de la DSM-5 (American Psychiatric Association 2013) ) es una enfermedad grave, recurrente e incapacitante, con una elevada prevalencia en nuestro medio (con estimaciones de la prevalencia-vida media de un 14,6% (Merikangas and Rihmer 2017), y prevalencia global en Europa en torno al 6-7% (Arias-de la Torre et al. 2023)), que ocasiona un deterioro de la salud médica general, el funcionamiento sociofamiliar, el rendimiento laboral y la calidad de vida. Se asocia a un incremento de morbimortalidad por riesgo de suicidio y otras causas médicas generales y psiquiátricas específicas asociadas (Akiskal 2017). Supone una de las causas mundiales más importantes de años de vida perdidos ajustados por discapacidad (DALYs) y la segunda causa global de años vividos con discapacidad (YLDs) (Hay et al. 2025).

Tanto el diagnóstico precoz como la instauración de un tratamiento antidepresivo eficaz son claves para el control de los síntomas, mejorar funcionalidad y evitar complicaciones. Sin embargo, existe una dificultad en la detección de la depresión ya desde los primeros contactos del paciente con el sistema sanitario, con una sensibilidad diagnóstica en atención primaria estimada en torno al 50%, de modo que aproximadamente la mitad de los casos no se detectan (Mitchell, Vaze, and Rao 2009), y es necesario un seguimiento continuado (con frecuencia demasiado prolongado) hasta el diagnóstico, lo que puede suponer un avance de la enfermedad y complicaciones médicas evitables.

El análisis de la voz podría suponer una ayuda en el diagnóstico de la depresión.

La posible utilidad clínica en depresión del análisis cuantitativo de algunas características vocales del discurso se demostró hace unos 50 años (Szabadi, Bradshaw, and Besson 1976), y el desarrollo de la Inteligencia Artificial y técnicas de Machine Learning ha facilitado la investigación en esta área en los últimos 15 años (Almaghrabi, Clark, and Baumert 2023; Briganti and Lechien 2025). Distintos modelos de aprendizaje automático de análisis acústico de la voz han conseguido sensibilidades y especificidades superiores al 70%, aunque con gran variabilidad en cuanto a la eficiencia relativa al tamaño muestral, desde el uso de fragmentos de discurso muy breves (tan breves como 25s) en poblaciones no clínicas muy amplias (más de 14.000 sujetos) (Mazur et al. 2025) hasta fragmentos más largos (de entre 7 y 50 min) en muestras más reducidas de pacientes diagnosticados de depresión (habitualmente entre 22 y 158 pacientes) (Lu et al. 2025), incluyendo contextos de riesgo de aislamiento y soledad no deseada como ocurre en poblaciones de edad avanzada (Martínez-Nicolás et al. 2025). Todos estos hallazgos, aunque con potencial aplicabilidad para el diseño de estudios piloto más homogéneos, generan una evidencia que aún adolece de sesgos de selección, diseños mayoritariamente transversales y carencias en la validación externa, lo que impide su traslación a la práctica clínica habitual en la actualidad (Briganti and Lechien 2025).

En resumen, aunque existe evidencia preliminar sugestiva de la consideración del análisis de la voz como un potencial biomarcador digital con utilidad clínica complementaria en el diagnóstico de la depresión, actualmente no existe suficiente evidencia que respalde su uso rutinario, por requerirse mayor validación en poblaciones diversas y una estandarización de la adquisición de datos del discurso, procesamiento y análisis (Almaghrabi, Clark, and Baumert 2023), además de diseños de seguimiento longitudinal más allá de asociaciones trasnversales.

Este protocolo aborda las carencias metodológicas mencionadas, proponiendo un marco longitudinal prospectivo en un entorno clínico que combina especificidad diagnóstica (población de pacientes diagnosticados de depresión moderada o grave) con amplitud de presentaciones (tanto de hospitalización como de consulta ambulatoria) y de tratamientos (incluyendo pacientes que reciban cualquier modalidad de tratamiento psicoterapéutico, farmacológico o de neuroestimulación, o incluso pacientes que deciden no recibir tratamiento, pero sí seguimiento con actitud expectante), para la recopilación y análisis de datos del habla en pacientes hispanohablantes con trastorno depresivo mayor y controles sanos. Al integrar características acústicas, paralingüísticas y lingüísticas y anclarlas a escalas clínicas validadas administradas por el médico, este protocolo pretende avanzar en la investigación del diagnóstico y seguimiento de la depresión asistido por el análisis de voz, pasando de la modelización exploratoria hacia una metodología clínicamente relevante y reproducible. Se trata de un estudio piloto exploratorio de validez de metodología en el contexto de un doctorado industrial.

**8.	HIPÓTESIS Y OBJETIVOS DE LA INVESTIGACIÓN**

**HIPÓTESIS**

El trastorno depresivo mayor y su evolución clínica se asocian a cambios en determinadas características de la voz y del discurso de tipo acústico, paralingüístico y lingüístico, que es posible analizar de un modo estandarizado y reproducible en un entorno de práctica clínica habitual.

**OBJETIVOS PRIMARIOS Y SECUNDARIOS.**

El objetivo general de este estudio es desarrollar y formalizar un protocolo estandarizado, reproducible y clínicamente fundamentado para la detección y monitorización longitudinal del trastorno depresivo mayor utilizando biomarcadores digitales derivados del habla, con un diseño que permita abordar las principales limitaciones de la evidencia actual: la heterogeneidad metodológica, la escasez de datos longitudinales y la infrarrepresentación de poblaciones clínicas no angloparlantes.

Objetivo primario:

El objetivo principal es establecer un marco clínico reproducible para la extracción y análisis de características acústicas, paralingüísticas y lingüísticas a partir de entrevistas clínicas semiestructuradas, permitiendo la discriminación entre pacientes con diagnóstico médico de trastorno depresivo mayor y controles sanos, utilizando modelos basados en la voz anclados en estándares de referencia clínicos validados (puntuaciones validadas de intensidad sintomática objetivados por el médico).

Objetivos secundarios:

1. Construcción de un corpus de habla clínica longitudinal en castellano, que comprenda grabaciones repetidas de voz de pacientes con trastorno depresivo mayor y grabaciones de una sola sesión de controles sanos.
2. Caracterizar la relación entre biomarcadores digitales derivados del habla y medidas de gravedad de la depresión valoradas por los médicos, incluyendo su evolución a lo largo del tiempo.
3. Evaluar el valor añadido de los enfoques multimodales que combinan características acústicas, paralingüísticas y lingüísticas en comparación con modelos unimodales.
4. Evaluar los cambios vocales intraindividuales a lo largo de múltiples puntos temporales de evaluación y su asociación con cambios en la gravedad clínica.
5. Proporcionar un marco metodológico transparente que pueda facilitar la replicación, la comparación cruzada y la validación externa futura en otras presentaciones clínicas y otros diagnósticos, lenguajes y entornos socioculturales.
6. Explorar si algún tipo de modalidad de tratamiento se asocia en mayor medida a los cambios en la voz con la evolución clínica.

**9.	MÉTODOS DE INVESTIGACIÓN**

**a) Diseño del estudio**:

Se trata de un estudio observacional, longitudinal prospectivo con grupo control realizado en un entorno clínico tanto ambulatorio como hospitalario. La duración total prevista del estudio es de 24 meses. Se incluirán dos tipos de participantes: (1) cohorte prospectiva de pacientes con diagnóstico médico de trastorno depresivo mayor (TDM) y (2) participantes como controles sanos sin antecedentes de trastornos psiquiátricos. Los pacientes con depresión recibirán 5 evaluaciones repetidas mensuales durante 4 meses, mientras que los participantes control serán evaluados una vez en condiciones idénticas de registro.

Los datos se obtendrán mediante la revisión de historias clínicas en el sistema de información de la CUN. Los investigadores del estudio garantizarán la confidencialidad de los datos de los sujetos y velarán porque se cumpla en todo momento con lo establecido en la ley 15/1999, de protección de datos de carácter personal y el RD 1720/2007.

**b) Entorno**:

Se ha optado por criterios de inclusión y exclusión amplios que permitan la inclusión de pacientes representativos de la práctica clínica real. La población del estudio consiste en pacientes con TDM con seguimiento en el departamento de Psiquiatría de la Clínica Universidad de Navarra, tanto en consulta ambulatoria como en régimen de hospitalización, independientemente de su tratamiento (psicoterapéutico, farmacológico o de neuroestimulación, o incluso pacientes que deciden no recibir tratamiento, pero sí seguimiento con actitud expectante), con los siguientes criterios de inclusión y sin ningún criterio de exclusión:

Criterios de inclusión/exclusión para los pacientes

Inclusión

1. Pacientes con edad igual o superior a 18 años,
2. De habla castellana (nativos o con fluidez suficiente para mantener una entrevista clínica)
3. Con capacidad para comprender los procedimientos del estudio y proporcionar consentimiento informado por escrito, según el juicio del investigador.
4. Con diagnóstico médico de depresión unipolar moderada o grave (con o sin síntomas psicóticos) según las categorías diagnósticas de CIE-10 (Episodio depresivo moderado (F32.1) o grave (F32.2 y F32.3), Trastorno depresivo recurrente episodio actual moderado (F33.1) o grave (F33.2 y F33.3)), independientemente de su tratamiento (psicoterapéutico, farmacológico o de neuroestimulación, o decisión de no recibir tratamiento).

Exclusión

1. Trastornos neurológicos graves que afectan a la producción del habla o a la función cognitiva (como demencia severa). Se permiten trastornos neurológicos no graves o demencias no severas.
2. Trastornos graves de la voz o del habla no relacionados con la depresión que impidan la realización de la entrevista clínica a través del lenguaje verbal.
3. Estado de intoxicación por consumo de sustancias en el momento de la evaluación.
4. Otras condiciones médicas graves que interfieran con la participación en este estudio o la producción del habla.

Criterios de inclusión/exclusión para los controles

Inclusión

1. Edad igual o superior a 18 años,

Exclusión

1. Antecedentes personales de depresión o cualquier otro trastorno psiquiátrico.
2. Trastornos neurológicos graves que afectan a la producción del habla o a la función cognitiva (como demencia severa). Se permiten trastornos neurológicos no graves o demencias no severas.
3. Trastornos graves de la voz o del habla no relacionados con la depresión que impidan la realización de la entrevista clínica a través del lenguaje verbal.
4. Estado de intoxicación por consumo de sustancias en el momento de la evaluación.
5. Otras condiciones médicas graves que interfieran con la participación en este estudio o la producción del habla.

**c) Selección de participantes, Visitas y Variables**:

**Selección de participantes**

Los pacientes serán invitados por los médicos del equipo investigador en su práctica clínica habitual y reclutados consecutivamente según los criterios de elegibilidad. Los controles serán invitados por los miembros del equipo investigador y en caso de aceptar serán remitidos a alguno de los médicos del equipo investigador para realizar la visita V0. Al tratarse de un estudio observacional, no se aplican procedimientos de aleatorización ni cegamiento. Todos los participantes se someterán a procedimientos idénticos de registro y evaluación dentro de sus respectivas cohortes.

**Calendario de visitas**

El estudio se llevará a cabo siguiendo una secuencia estructurada de visitas clínicas diseñadas para garantizar la recogida de datos estandarizada minimizando la carga para los participantes. Todos los procedimientos del estudio serán realizados por personal médico cualificado en un entorno de consulta ambulatoria o de hospitalización.

**Visita de reclutamiento, consentimiento y evaluación inicial (Visita basal, V0)**. Los posibles participantes serán informados sobre los objetivos, el alcance y la metodología del estudio sobre detección de depresión mediante análisis de voz y transcripción de voz. Se explicará explícitamente la naturaleza no invasiva del procedimiento, consistente en la audio grabación de una parte específica de la entrevista médica, la confidencialidad de los datos recopilados y el uso exclusivo de los datos con fines de investigación. A los participantes que muestren interés en participar en el estudio se les proporcionará la Hoja de Información del Participante y el formulario de consentimiento informado, y se les dará tiempo suficiente para leer la documentación y hacer preguntas.

Se obtendrá el consentimiento informado por escrito antes de iniciar cualquier procedimiento relacionado con el estudio. A continuación, se realizará una entrevista clínica estructurada para recopilar información sociodemográfica y clínica básica, seguida de la evaluación psicométrica inicial utilizando las escalas clínicas definidas en el protocolo. Durante la misma sesión, se obtendrán grabaciones de voz bajo condiciones controladas siguiendo un protocolo estandarizado que incluye una combinación de preguntas estructuradas y abiertas. Estas grabaciones constituirán la base para la posterior extracción de variables acústicas, paralingüísticas y lingüísticas.

Los controles sanos sólo recibirán la visita V0. Sólo los pacientes recibirán visitas de seguimiento.

**Visitas de seguimiento (Visitas V1, V2, V3 y V4).** Los pacientes con trastorno depresivo mayor serán invitados a asistir a cuatro visitas de seguimiento, coincidentes con las visitas médicas programadas de su seguimiento por práctica clínica habitual, aproximadamente cada 4 semanas, con una ventana permitida de ±7 días. En cada visita de seguimiento, las grabaciones de la voz y del discurso se repetirán utilizando el mismo protocolo estandarizado aplicado al inicio, lo que permitirá la evaluación longitudinal de los cambios intraindividuales en variables derivadas del habla. Además, se actualizarán medidas de gravedad depresiva tanto objetivadas por los médicos como autoinformadas por los pacientes, y se documentará cualquier cambio clínico relevante, incluidos ajustes del tratamiento o eventos adversos.

Los datos recogidos en las visitas de seguimiento permitirán analizar las trayectorias temporales de las características acústicas, paralingüísticas y del discurso en relación con cambios en la sintomatología depresiva, apoyando enfoques de modelización tanto transversal como longitudinal.

**Variables de exposición:**

-Diagnóstico médico de depresión unipolar moderada o grave (con o sin síntomas psicóticos) según las categorías diagnósticas de CIE-10 (Episodio depresivo moderado (F32.1) o grave (F32.2 y F32.3), Trastorno depresivo recurrente episodio actual moderado (F33.1) o grave (F33.2 y F33.3)), independientemente de su tratamiento (psicoterapéutico, farmacológico o de neuroestimulación, o decisión de no recibir tratamiento).

**De resultados primarios:**

-Protocolo de grabación de voz y variables del análisis multimodal de la voz y del discurso (V0, V1 a los 30 (+/-7) días, V2 a los 60 (+/-7) días, V3 a los 90 (+/-7) días y V4 a los 120 (+/-7) días).

Protocolo de grabación de voz

Se grabará exclusivamente la parte de la entrevista médica correspondiente a la escala MADRS (entre 10 y 30min), que incluirá una parte de discurso libre inicial. Las grabaciones de voz se obtendrán usando un micrófono Jabra Speak 510 USB omnidireccional. El micrófono se colocará a una distancia de no más de 20 cm desde la boca del participante. Las grabaciones se realizarán en un ambiente hospitalario tranquilo (consulta). Al evitar intencionadamente un micrófono de estudio de alta gama, se pretende maximizar la validez ecológica y facilitar una posible aplicabilidad en la práctica clínica habitual. Este enfoque es coherente con estudios previos en el campo, que han empleado dispositivos de grabación no especializados (Menne et al. 2024; Wasserzug et al. 2023). Las grabaciones de audio se almacenarán en formato WAV sin comprimir para preservar la señal. Todos los archivos estarán etiquetados de con códigos alfanuméricos seudonimizados. Durante el preprocesado, se eliminará el discurso del entrevistador y sólo se utilizará la señal del habla del participante para el análisis.

Variables del análisis multimodal de la voz y del discurso. Las variables incluidas en este estudio se han seleccionado a partir de la evidencia de estudios previos:

1. Acústicas:
   1.1. Tono (media F0, desviación estándar, Rango): Frecuencia en Hz.
   1.2. Energía / intensidad (media, desviación estándar): dB.
   1.3. MFCCs (coeficientes 1–13, media / estándar desviación): Coeficientes cepstrales.
   1.4. Relación armónico-ruido (HNR) en dB.
   1.5. Fluctuación (Jitter) (%).
   1.6. Brillo (shimmer) (%).
   1.7. Descriptores espectrales basados en frecuencia con centroide espectral / desaceleración / pendiente (Hz o unidades normalizadas).
   1.8. Formantes (media / ancho de banda F1–F3), frecuencia (Hz) y ancho de banda (Hz).
   1.9. Entropía espectral / entropía prosódica: Índice de entropía (adimensional).
   1.10. Características acústicas basadas en incrustaciones (por ejemplo, Wav2Vec2, HuBERT) (representaciones vectoriales de alta dimensión).
2. Paralinguísticas
   2.1. Tasa de habla / velocidad de articulación / duración de pausa: Palabras por segundo (p/s); Duración de la pausa en segundos (s).
   2.2. Rango prosódico (F0 y variabilidad de intensidad): Rango (Hz, dB) o coeficiente de variación (%).
   2.3. Razón de pausas (silencio / habla): Proporción (0–1) o porcentaje (%).
3. Linguísticas
   3.1. Polaridad del sentimiento (media por enunciado): Escala continua de −1 (negativo) a +1 (positivo).
   3.2. Proporción emocional negativa de palabras: Porcentaje (%) de palabras con valencia negativa.
   3.3. Pronombres de primera persona del singular: Porcentaje (%) o frecuencia por cada 100 palabras.
   3.4. Diversidad léxica (MTLD, Honoré, TTR): Índices de diversidad léxica (adimensional).
   3.5. Recuento de palabras/longitud media de las frases: Número de palabras; palabras por frase.
   3.6. Varianza léxica del sentimiento (intrafrase): desviación estándar de las puntuaciones de sentimiento (−1 a +1).
   3.7. Coherencia / cohesión léxica (similitud de incrustación BERT): Puntuación de similitud coseno (0–1).

-Intensidad de síntomas depresivos objetivada por el médico (V0, V1 a los 30 (+/-7) días, V2 a los 60 (+/-7) días, V3 a los 90 (+/-7) días y V4 a los 120 (+/-7) días), a través de la escala Montgomery-Asberg para la depresión (MADRS) (Montgomery and Asberg 1979; Lobo et al. 2002) (escala clínica realizada por el médico).

-Gravedad clínica global objetivada por el médico (V0, V1 a los 30 (+/-7) días, V2 a los 60 (+/-7) días, V3 a los 90 (+/-7) días y V4 a los 120 (+/-7) días), a través de la escala de Impresión Clínica Global (ICG) (Guy 1976)(escala clínica realizada por el médico).

**De resultados secundarios:**

-Diagnóstico principal (basal): tipo de trastorno depresivo mayor unipolar.

-Gravedad de los síntomas depresivos referida por el paciente (V0, V1 a los 30 (+/-7) días, V2 a los 60 (+/-7) días y V3 a los 90 (+/-7) días y V4 a los 120 (+/-7) días), a través del Cuestionario de Salud del Paciente – 9 (PHQ-9) (cuestionario autoaplicado).

-Comorbilidades psiquiátricas (incluyendo antecedentes de autolesiones e ideación y conducta suicida, duración del episodio actual, tiempo desde el diagnóstico inicial y número de episodios previos) y médicas generales, con comedicaciones y, si se dispone de ellas, isoformas CYP o farmacodinámicas (basal)

-Variables antropométricas, demográficas y hábitos de vida (basal): Sexo, edad, estado civil, lugar de residencia, lugar en la fratria, talla, peso, IMC, práctica de ejercicio físico (intensidad, frecuencia), dieta (saludable/no saludable), grado de formación escolar, profesional o académica, actividad de cuidado familiar (cuidado de hijos o familiares dependientes), actividad laboral (sí/no, tipo), relaciones sociales, creencia religiosa (si/no), práctica religiosa comunitaria (frecuencia).

-Variables de tratamiento farmacológico (V0, V1 a los 30 (+/-7) días, V2 a los 60 (+/-7) días, V3 a los 90 (+/-7) días y V4 a los 120 (+/-7) días): Dosis, intervalo de dosificación, hora de administración, duración de tratamiento (para cada fármaco que reciba el paciente).

-Variables de tratamiento no farmacológico (V0, V1 a los 30 (+/-7) días, V2 a los 60 (+/-7) días, V3 a los 90 (+/-7) días y V4 a los 120 (+/-7) días): Medidas higiénico-dietéticas de estilo de vida (higiene del sueño, patrón de dieta saludable, ejercicio físico regular, abandono de tabaco y/o tóxicos). Tipo de psicoterapia, Cantidad/frecuencia de sesiones de psicoterapia, estimulación magnética transcraneal repetitiva (tipo y frecuencia); terapia electroconvulsiva (tipo y frecuencia).

-Otras variables (basal): antecedentes psiquiátricos familiares, acontecimientos vitales estresantes-precipitantes/traumáticos actuales y previos, hábitos tóxicos y tipo de tóxicos consumidos

d) Fuentes de datos:

La información de las variables para cada participante se recogerá durante cada visita en un cuaderno de recogida de datos seudonimizado.

**e) Tamaño muestral**

El estudio se plantea con una muestra total de 75 sujetos, compuesta por 25 pacientes con depresión y 50 controles sanos, lo que constituye un diseño clínicamente realista y coherente con la capacidad de reclutamiento disponible.

La muestra presenta una distribución no balanceada entre grupos, circunstancia que se ha tenido en cuenta en el planteamiento metodológico y en la interpretación de los resultados. Esta configuración permite abordar el estudio desde una doble perspectiva. Por un lado, hace posible la comparación entre pacientes con depresión y controles sanos, con el fin de explorar la existencia de diferencias vocales asociadas al trastorno. Por otro, el diseño incorpora un componente longitudinal en el grupo clínico, que permite analizar la evolución intraindividuo a lo largo del seguimiento y valorar el potencial de la voz no solo como herramienta de detección, sino también de monitorización. De este modo, aunque la muestra no sea simétrica entre grupos, su estructura permite extraer información complementaria y maximizar el valor científico de los datos disponibles dentro de un contexto clínico realista.

Aunque el número de pacientes con depresión es limitado, el valor añadido del estudio reside en su componente longitudinal, ya que estos 25 pacientes serán evaluados en 5 visitas (basal y 4 de seguimiento), generando 125 registros en el grupo clínico y permitiendo no solo comparar pacientes y controles, sino también analizar la evolución intraindividuo de los biomarcadores vocales en relación con el curso clínico.

No obstante, dado que las 5 observaciones de cada paciente con depresión no son independientes entre sí, sino que están correlacionadas, el número bruto de registros no puede interpretarse como tamaño muestral efectivo. Para corregir este hecho se considera el efecto del diseño asociado a medidas repetidas, calculado mediante la expresión DEFF=1+(m-1)ICC, donde m es el número de medidas por sujeto y ICC es el coeficiente de correlación intraclase asumido entre visitas. En este caso, tomando m=5 y asumiendo una correlación moderada de ICC=0.2, se obtiene: DEFF=1+(5-1)⋅0.2=1+0.8=1.8. Por tanto, el tamaño efectivo del grupo con depresión puede aproximarse como n_ef_ =125/1.8=69.4. Sumando este valor a los 50 controles independientes, el tamaño efectivo total del estudio sería aproximadamente 69.4+50=119.4 observaciones efectivas.

**f) Gestión de los datos**.

En cuanto a datos clínicos, sólo tendrán acceso a los datos de la historia clínica y cuaderno de recogida de datos necesarios para alcanzar los objetivos del proyecto las personas designadas por el promotor, el monitor, el investigador y su equipo de colaboradores y las autoridades sanitarias pertinentes.

En cuanto a grabaciones de voz pseudonimizadas, se trasladarán íntegramente y sin distorsionar, físicamente desde un dispositivo de memoria externa a los servidores de NNBi, donde se almacenarán hasta el 31/12/2029, destruyéndose esa fecha como máximo, una vez concluido este proyecto. La empresa NNBi se compromete a realizar la destrucción de todas las grabaciones y sus copias de seguridad antes del 01/01/2030. Los servidores de NNBi destinados al almacenamiento de los audios anonimizados garantizan la seguridad, confidencialidad, integridad y disponibilidad de los datos conforme a la normativa vigente. NNBi está certificada en el Esquema Nacional de Seguridad (ENS) en nivel Alto y en la norma ISO 27001, lo que acredita un marco de seguridad alineado con las mejores prácticas internacionales. En este contexto, se aplican medidas técnicas y organizativas avanzadas: cifrado de la información en reposo y en tránsito, control de acceso estricto basado en roles y principio de mínimo privilegio, autenticación robusta, segregación de entornos, y clasificación de la información según su sensibilidad. Además, se asegura la monitorización y registro de accesos y operaciones para detectar usos indebidos, junto con mecanismos de protección de integridad y prevención de alteraciones no autorizadas. Finalmente, la gestión del ciclo de vida de la información incluye copias de seguridad cifradas, procedimientos de recuperación ante incidentes y borrado seguro al finalizar el periodo de conservación definido, minimizando el riesgo para los pacientes y asegurando un tratamiento responsable de los datos.

El promotor y los investigadores del estudio deben garantizar la confidencialidad de los datos de los sujetos y velar porque se cumpla en todo momento con lo establecido en la ley 3/2018, de protección de datos de carácter personal y el Reglamento (UE) 2016/679 del Parlamento europeo y del Consejo de 27 de abril de 2016 de Protección de Datos.

**g) Análisis de los datos.**

Comprende los siguientes análisis estadísticos y estrategia de modelización:

Se realizará un análisis descriptivo inicial de todas las variables extraídas. Para las variables cuantitativas (acústicas, paralingüísticas y lingüísticas) se calculará la media y la desviación estándar, mientras que para las variables cualitativas se utilizarán porcentajes y frecuencias.

Para el cumplimiento de los objetivos del estudio, el análisis se estructurará en tres ejes:

1.	Asociación y Discriminación Diagnóstica: Se emplearán coeficientes de correlación (Pearson/Spearman) para valorar la asociación entre los biomarcadores continuos y las escalas de intensidad sintomática (MADRS, PHQ-9 y CGI). Para la discriminación entre pacientes y controles sanos, se utilizarán modelos de clasificación. Se reportará el Área Bajo la Curva (AUC) como métrica principal de rendimiento, junto con la Odds Ratio y sus intervalos de confianza al 95%.

2.	Evaluación Multimodal: Se realizará una comparación sistemática entre modelos unimodales (basados exclusivamente en audio o texto) y modelos de fusión multimodal. Para el procesamiento de las transcripciones y la extracción de características semánticas complejas, se contempla el uso de modelos de lenguaje avanzados. Este análisis permitirá cuantificar el valor añadido de combinar características acústicas, paralingüísticas y lingüísticas en la predicción de la gravedad clínica.

3.	Análisis Longitudinal e Intra-individual: Para evaluar la evolución de los biomarcadores a lo largo del tiempo y su relación con el cambio en la severidad clínica, se emplearán modelos de efectos mixtos o modelos de diferencias finitas. Estos modelos permiten gestionar la correlación intra-sujeto debida a las medidas repetidas y controlar la variabilidad inter-individual.

Validación y Transparencia: Todas las evaluaciones se realizarán mediante una estrategia de división de datos a nivel de participante (Leave-One-Group-Out), asegurando que todas las visitas de un mismo sujeto pertenezcan exclusivamente al grupo de entrenamiento o al de prueba para evitar sesgos de sobreajuste. Este aspecto es metodológicamente relevante por la necesidad de utilizar un conjunto de datos para entrenamiento del modelo y otro conjunto de datos para validar los resultados. En ambos grupos se incluirán tanto pacientes como controles sanos.

Para garantizar la interpretación clínica de los resultados, se realizarán análisis de contribución de características (atribución basada en valores de Shapley o SHAP), permitiendo identificar qué señales específicas (p. ej., pausas, variaciones de tono o contenido léxico) impulsan las predicciones del modelo. Los análisis se realizarán utilizando software estadístico especializado (Python v3.x). Los valores p < 0.05 serán considerados estadísticamente significativos.

**h) Control de calidad.**

El control de los datos recogidos en el CRD estará asegurado por propio equipo investigador. El investigador principal controlará el seguimiento del protocolo del estudio.

**i) Limitaciones de los métodos de investigación.**

Deben reconocerse las siguientes limitaciones y desafíos anticipados del protocolo propuesto, especialmente aquellos inherentes a los estudios clínicos basados en la voz que buscan equilibrar la estandarización, la validez ecológica y la viabilidad traslacional:

1) En primer lugar, las escalas clínicas incluidas (MADRS, ICG y PHQ-9) se utilizan como estándares de referencia principales de intensidad sintomática y gravedad, pero están sujetas a variabilidad en la medición y carecen de validez diagnóstica. Es muy importante enfatizar que el protocolo actual no pretende sustituir el juicio clínico diagnóstico, sino complementarlo identificando marcadores derivados del habla que se correlacionan con medidas establecidas de gravedad depresiva.
2) Aunque las grabaciones del habla se obtienen en condiciones de un entorno clínico controlado (consulta, planta de hospitalización), se espera una variabilidad significativa entre individuos en el estilo de habla, la expresividad emocional y el compromiso durante las entrevistas clínicas. Para equilibrar la estandarización con las condiciones clínicas de mundo real, se utiliza un protocolo de entrevista semiestructurado administrado por el médico, que contempla un espacio un discurso libre y espontáneo del paciente, para provocar un discurso naturalista. Esto mejora la validez ecológica y la relevancia clínica, pero puede introducir variabilidad en el contenido del habla, la duración y la cobertura temática, lo que a su vez puede afectar tanto a las mediciones acústicas como lingüísticas.
3) La recogida de datos longitudinales presenta desafíos prácticos. Las evaluaciones repetidas aumentan la carga de los participantes y pueden llevar a visitas perdidas o a datos de seguimiento incompletos. Para mitigar este riesgo, las visitas de seguimiento se programan dentro de plazos flexibles y todos los procedimientos están diseñados para ser breves, muy adaptados a la práctica clínica habitual y con una carga de esfuerzo añadida mínima para el paciente. No obstante, se prevé cierto grado de desgaste y se abordará explícitamente en la estrategia analítica.
4) El tamaño de la muestra planificada sigue siendo moderado. Aunque la estructura longitudinal proporciona información valiosa sobre trayectorias vocales intraindividuales, una muestra moderada puede limitar el poder estadístico, aumentar la incertidumbre en las estimaciones de rendimiento, restringir la exploración de efectos de interacción complejos y aumentar el riesgo de sobreajuste, especialmente en modelos de mayor dimensión o multimodales.
5) La variabilidad técnica en las condiciones de grabación como el ruido de fondo o la posición del micrófono puede influir en las mediciones acústicas. El protocolo aborda esto utilizando un dispositivo de registro estandarizado y su colocación, evitando intencionadamente equipos de alto nivel o de laboratorio para maximizar la aplicabilidad clínica. Este compromiso prioriza la aplicabilidad real sobre la fidelidad máxima de la señal y puede reducir la relación señal-ruido para ciertos biomarcadores acústicos.
6) El estudio se realiza en un único centro clínico y exclusivamente en participantes hispanohablantes, lo que puede restringir la validez externa en sistemas sanitarios que atienden a poblaciones con dialectos u otros contextos lingüísticos. Sin embargo, este enfoque también aborda una laguna relevante en la literatura, que se ha centrado predominantemente en conjuntos de datos en inglés.
7) Por último, la exclusión de ciertas características de alta complejidad, como el uso obligatorio de incrustaciones acústicas, puede limitar el rendimiento predictivo máximo. Sin embargo, esta decisión es coherente con el énfasis del estudio en la reproducibilidad metodológica, la interpretabilidad y el despliegue clínico, y permite que el conjunto de características principales siga siendo transparente, auditable y factible de calcular en entornos hospitalarios rutinarios.

**10.	PROTECCIÓN DE LAS PERSONAS SOMETIDAS AL ESTUDIO**

**a) Evaluación beneficio-riesgo para los sujetos de investigación, en su caso.**

No procede, ya que en este estudio se analizan variables recogidas en la historia clínica por práctica clínica habitual, cuestionarios clínicos especificados en variables y consentimiento para grabación de una parte de la entrevista médica.

**b) Consideraciones sobre información a los sujetos y consentimiento informado.**

Una vez explicado el estudio en su totalidad al paciente, se obtendrá su consentimiento informado por escrito o bien de su tutor o representante legal antes de hacer efectiva su participación en el estudio.

El investigador (o persona delegada) firmará y fechará también el formulario de consentimiento. El investigador archivará el Formulario de Consentimiento original firmado en el Archivo del Investigador del centro.

El paciente recibirá el Formulario de Consentimiento Informado y se le informará de que la participación en el estudio es voluntaria y puede retirarse en cualquier momento sin perjuicio de su asistencia médica posterior. Ni la Hoja de Información del Paciente ni el Consentimiento Informado pueden modificarse sin el acuerdo del Comité de Ética de la investigación con medicamentos y del promotor.

Un ejemplar del Formulario de Consentimiento Informado debe estar en poder del paciente.

El Formulario de Consentimiento incluye la información relativa a la necesidad de acceder las Historias Clínicas.

**c) Confidencialidad de los datos:**

Con el fin de garantizar la confidencialidad de los datos; se identificará a los pacientes del estudio con un número, de forma correlativa según el orden de inclusión. Los cuadernos de recogida de datos (CRD), informes y comunicaciones del estudio irán identificados con dicho número. Sólo tendrán acceso al material identificado necesario para alcanzar los objetivos del estudio el promotor, el investigador y su equipo de colaboradores, el Comité de Ética de la Investigación con medicamentos que tutela el estudio y las autoridades sanitarias pertinentes, según autorización del paciente.

El contenido de los CRD así como los documentos generados durante el estudio serán custodiados de usos no permitidos por personas ajenas al estudio, y por tanto, serán considerados estrictamente confidenciales y no serán revelados a terceros excepto los especificados en el párrafo anterior.

El promotor y los investigadores del estudio deben garantizar la confidencialidad de los datos de los sujetos y velar porque se cumpla en todo momento con lo establecido en la ley 3/2018, de protección de datos de carácter personal y el reglamento europeo (UE) 2016/679.

**d) Interferencia con los hábitos de prescripción del médico**

La realización de este estudio prospectivo no modifica los hábitos de prescripción médica ya que se incluyen pacientes independientemente de su tratamiento (psicoterapéutico, farmacológico o de neuroestimulación), e incluso pacientes que deciden no recibir tratamiento, pero sí seguimiento con actitud expectante. Como objetivo secundario, se explorará si algún tipo de modalidad de tratamiento se asocia en mayor medida a los cambios en la voz con la evolución clínica.

**11.	GESTIÓN Y NOTIFICACIÓN DE REACCIONES ADVERSAS y demás eventos relevantes que** **aparezcan durante el estudio**

Los casos de sospechas de reacciones adversas graves e inesperadas se comunicarán por el cauce oficial previsto en la CUN.

DEFINICIONES

Acontecimiento adverso se define como cualquier incidencia perjudicial para la salud en un paciente o sujeto de un estudio clínico tratado con un medicamento, aunque no tenga necesariamente una relación causal con dicho tratamiento.

Un AA puede ser, por tanto, cualquier signo desfavorable y no intencionado (incluyendo un hallazgo anormal de laboratorio), síntoma o enfermedad temporalmente asociada con el uso de un medicamento en investigación, esté o no relacionado con el medicamento en investigación.

Acontecimiento adverso grave se define como cualquier acontecimiento adverso o reacción adversa que, a cualquier dosis:

- Provoque la muerte del paciente.
- Amenace la vida del paciente (en opinión del investigador, el paciente en el momento del AA o RA está en un riesgo real de muerte; no se refiere a que el AA/RA hipotéticamente pudiera haber ocasionado la muerte en el caso de haber sido más intenso).
- Requiera la hospitalización o prolongación de la hospitalización del paciente
- Provoque invalidez o incapacidad permanente o importante
- Dé lugar a una anomalía o malformación congénita

Reacción adversa se considera a cualquier reacción nociva y no intencionada a un medicamento en investigación, independientemente de la dosis administrada. A diferencia de un AA, en el caso de una reacción adversa existe una sospecha de relación causal entre el medicamento en investigación y el acontecimiento adverso.

La determinación de la posible relación con el tratamiento del estudio deberá realizarse de acuerdo con las siguientes definiciones:

- NO RELACIONADO: No hay evidencia de ninguna relación causal.
- IMPROBABLE: Existe poca evidencia que sugiera una relación causal (p.ej. el acontecimiento no se presentó en un período de tiempo razonable después de la administración del medicamento/procedimiento del estudio). Hay otra explicación razonable para el acontecimiento (p.ej. la condición clínica del paciente, otros tratamientos concomitantes).
- POSIBLE: Existen evidencias que sugieren una posible relación causal (p.ej. porque el acontecimiento ocurrió en un tiempo razonable después de la administración del medicamento del estudio). Sin embargo, la influencia de otros factores puede haber contribuido al acontecimiento (p.ej. la condición clínica del paciente, otros tratamientos concomitantes).
- PROBABLE: Existen evidencias que sugieren una relación causal y la influencia de otros factores es poco probable.
- DEFINITIVO: Existen evidencias claras que sugieren una relación causal y se puede descartar una posible contribución de otros factores.

Reacción Adversa Grave e Inesperada (RAGI). Cualquier reacción adversa grave cuya naturaleza, intensidad o consecuencias no se corresponde con la información de referencia para el medicamento.

El carácter inesperado de una reacción adversa se basa en el hecho de no haber sido observado previamente y no se basará en lo que pudiera ser anticipado en función de las propiedades farmacológicas del medicamento.

COMUNICACIÓN DE ACONTECIMIENTOS ADVERSOS.

Los casos de reacciones adversas graves e inesperadas deben se registrados sistemáticamente por el profesional sanitario y transmitidos a las autoridades por el cauce oficial establecido para ello en la CUN. Este estudio no modifica la pauta terapéutica de los participantes, lo cual incluye las obligaciones inherentes a la administración de medicamentos y la detección y comunicación de los mismos a los sistemas de farmacovigilancia cuando proceda.

El profesional responsable del tratamiento será el encargado de comunicar las sospechas de reacciones adversas cuando proceda a través del cauce habituales establecido para ello en la CUN.

**12.	PLAN DE TRABAJO**

**(tareas, hitos y cronología** del estudio).

| **Actividad** | **Fecha inicio** | **Fecha fin** |
| --- | --- | --- |
| Recogida de datos | mayo 2026 | diciembre 2027 |
| Análisis e interpretación de los datos obtenidos | enero 2028 | Febrero 2028 |
| Redacción | Marzo 2028 | Junio 2028 |
| Informe final de resultados | Septiembre 2028 |  |

**13.	PLANES DE DIFUSIÓN Y COMUNICACIÓN DE LOS RESULTADOS DEL ESTUDIO.**

Los resultados se enviarán para su publicación a revistas científicas especializadas con revisión por pares.

**14.	REFERENCIAS.**

Akiskal, Hagop S. 2017. “13.4 Mood Disorders: Clinical Features.” In *Kaplan and Sadock’s Comprehensive Textbook of Psychiatry, Tenth Edition*, edited by BJ Sadock, VA Sadock, and P Ruiz, 1630–60.

Almaghrabi, Shaykhah A., Scott R. Clark, and Mathias Baumert. 2023. “Bio-Acoustic Features of Depression: A Review.” *Biomedical Signal Processing and Control* 85 (August):105020. https://doi.org/10.1016/J.BSPC.2023.105020.

American Psychiatric Association. 2013. “Diagnostic and Statistical Manual of Mental Disorders (5th Ed.) (DSM–5).”

Arias-de la Torre, Jorge, Gemma Vilagut, Amy Ronaldson, Ioannis Bakolis, Alex Dregan, Vicente Martín, Gonzalo Martinez-Alés, et al. 2023. “Prevalence and Variability of Depressive Symptoms in Europe: Update Using Representative Data from the Second and Third Waves of the European Health Interview Survey (EHIS-2 and EHIS-3).” *The Lancet Public Health* 8 (11): e889–98. https://doi.org/10.1016/S2468-2667(23)00220-7.

Briganti, Giovanni, and Jérôme R. Lechien. 2025. “Speech and Voice Quality as Digital Biomarkers in Depression: A Systematic Review.” *Journal of Voice*. https://doi.org/10.1016/j.jvoice.2025.05.002.

García-Portilla, M.P., M.T. Fernández-Bascarán, P.A. Sáiz-Martínez, M.T. Bobes-Bascarán, M. Bousoño-García, and J. Bobes-García. 2022. *Banco de Instrumentos Básicos Para La Práctica de La Psiquiatría Clinica*. Octava ed.

Guy, W. 1976. *Early Clinical Drug Evaluation (ECDEU) Assessment Manual*. Rockville, Maryland: National Institute of Mental Health.

Hay, Simon I., Kanyin Liane Ong, Damian F. Santomauro, A. Bhoomadevi, Mohammad Amin Aalipour, Hasan Aalruz, Hazim S. Ababneh, et al. 2025. “Burden of 375 Diseases and Injuries, Risk-Attributable Burden of 88 Risk Factors, and Healthy Life Expectancy in 204 Countries and Territories, Including 660 Subnational Locations, 1990–2023: A Systematic Analysis for the Global Burden of Disease Study 2….” *The Lancet* 406 (10513): 1873–1922. https://doi.org/10.1016/S0140-6736(25)01637-X.

Lobo, Antonio, Lorenzo Chamorro, Antonio Luque, Rafael Dal-Ré, Xavier Badia, Eva Baró, Concepción Lacámara, et al. 2002. “[Validation of the Spanish Versions of the Montgomery-Asberg Depression and Hamilton Anxiety Rating Scales].” *Medicina Clinica* 118 (13): 493–99. https://doi.org/10.1016/S0025-7753(02)72429-9.

Lu, Wei, Xiaowei Tang, Chuan Huang, Man Wei, Chengxin Bai, Xuqing Fan, and Dongmei Wu. 2025. “Diagnostic Accuracy of Traditional and Deep Learning Methods for Detecting Depression Based on Speech Features: A Systematic Review and Meta-Analysis.” *BMC Psychiatry* 25 (1). https://doi.org/10.1186/S12888-025-07628-Z.

Martínez-Nicolás, Israel, Diego Criado, Fernando Gordillo, Francisco Martínez-Sánchez, and Juan José G. Meilán. 2025. “Speech Analysis for Detecting Depression in Older Adults: A Systematic Review.” *Frontiers in Psychology* 16. https://doi.org/10.3389/FPSYG.2025.1715538.

Mazur, Alexa, Harrison Costantino, Prentice Tom, Michael P. Wilson, and Ronald G. Thompson. 2025. “Evaluation of an AI-Based Voice Biomarker Tool to Detect Signals Consistent With Moderate to Severe Depression.” *Annals of Family Medicine* 23 (1): 60–65. https://doi.org/10.1370/AFM.240091.

Menne, Felix, Felix Dörr, Julia Schräder, Johannes Tröger, Ute Habel, Alexandra König, and Lisa Wagels. 2024. “The Voice of Depression: Speech Features as Biomarkers for Major Depressive Disorder.” *BMC Psychiatry* 24 (1): 794. https://doi.org/10.1186/s12888-024-06253-6.

Merikangas, KR, and Z Rihmer. 2017. “13.2 Mood Disorders: Epidemiology.” In *Kaplan and Sadock’s Comprehensive Textbook of Psychiatry, Tenth Edition*, 1614–19.

Mitchell, Alex J., Amol Vaze, and Sanjay Rao. 2009. “Clinical Diagnosis of Depression in Primary Care: A Meta-Analysis.” *The Lancet* 374 (9690): 609–19. https://doi.org/10.1016/S0140-6736(09)60879-5.

Montgomery, S. A., and M. Asberg. 1979. “A New Depression Scale Designed to Be Sensitive to Change.” *British Journal of Psychiatry* 134 (4): 382–89. https://doi.org/10.1192/BJP.134.4.382,.

Szabadi, E., C. M. Bradshaw, and J. A.O. Besson. 1976. “Elongation of Pause-Time in Speech: A Simple, Objective Measure of Motor Retardation in Depression.” *The British Journal of Psychiatry* 129 (6): 592–97. https://doi.org/10.1192/BJP.129.6.592.

Wasserzug, Yael, Yoav Degani, Mili Bar-Shaked, Milana Binyamin, Amit Klein, Shani Hershko, and Yechiel Levkovitch. 2023. “Development and Validation of a Machine Learning-Based Vocal Predictive Model for Major Depressive Disorder.” *Journal of Affective Disorders* 325 (March):627–32. https://doi.org/10.1016/j.jad.2022.12.117.

World Health Organization. 2016. “ICD-10 : International Statistical Classification of Diseases and Related Health Problems : Tenth Revision, 2nd Ed.”
